# Unraveling the multifaceted impact of tuberculosis on coinfections

**DOI:** 10.1016/j.isci.2026.117133

**Published:** 2026-08-12

**Authors:** Joan Fine, François Trottein, Arnaud Machelart, Valentin Sencio

**Affiliations:** 1 Univ. Lille, CNRS, INSERM, CHU Lille, Institut Pasteur de Lille, U1019, UMR 9017 CIIL – Center for Infection and Immunity of Lille, Lille, France

**Keywords:** tuberculosis, coinfection, lungs, inflammation, host-pathogen interaction

## Abstract

Tuberculosis (TB) remains the leading cause of death worldwide from a single bacterial agent. However, an estimated one-quarter of the world’s population carries an asymptomatic form of TB. In this state, the host maintains a chronic inflammatory lung environment in which the infection is contained and may even be cleared. TB can be lifelong; thus, patients are likely to contract superinfection by another pathogen. It is therefore relevant to investigate the impact of *Mycobacterium tuberculosis* (*Mtb*), the bacterium responsible for TB, on the host’s capacity to cope with a second infectious agent. TB is increasingly recognized as a dynamic disease that exists along a broad clinical and immunological spectrum, far beyond the conventional dichotomy of active and latent infection. Yet, in the context of coinfections, this complexity is often overlooked. This review therefore bridges historical outbreaks, global epidemiology, and experimental models to illustrate the interplay between TB and co-occurring infections. In particular, *Mtb* infection shapes the outcome of secondary infections, while coinfecting pathogens can in turn reshape TB progression, immune control, and disease severity.

## Graphical abstract

**Figure:**
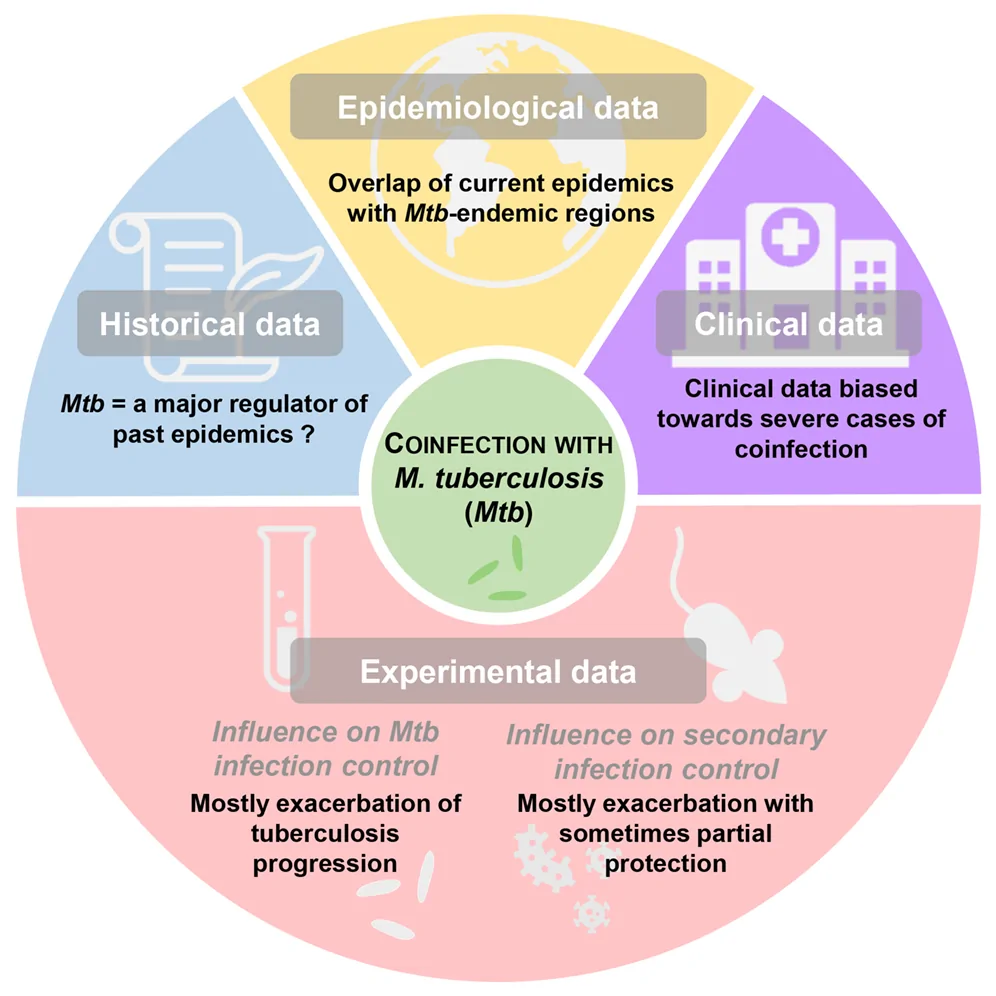

## Teaser

Health sciences; Medicine; Medical specialty; Medical microbiology

## Introduction

Studies of host susceptibility to infectious agents typically employ single-pathogen infection models to minimize biological noise. Yet this reductionist strategy ignores a fundamental principle that the immune landscape is never static. Host defense capacity is shaped dynamically by age, sex, nutritional and allergic status, comorbidities, vaccination history, and crucially, concurrent infections. These factors collectively dictate disease outcomes in natural settings.

Coinfection involving two or more distinct pathogens can bidirectionally alter the host’s ability to control each infection independently. It affects the immunological status of the host through various mechanisms, including tissue damage, quantitative alterations of the immune response (ranging from hyperinflammation to immune suppression), as well as functional dysregulation of protective immune pathways, or changes in host cell metabolism (for review^1^). In the literature, superinfections are often described as exacerbations of one or the other pathology, but cases of protection during coinfection have also been reported. Predicting how one infection affects the other is essential for adapting patient treatment and/or identifying new therapeutic strategies.

With 10.8 million new cases each year and 1.25 million deaths (including 1.09 million among human immunodeficiency virus (HIV)-negative people), tuberculosis (TB) is the world’s leading cause of death from a single infectious agent.^2^ It is caused by *Mycobacterium tuberculosis* (*Mtb*), an aerosol-borne bacterium. The bacillus infects lung cells, mainly alveolar macrophages, where it modifies endocytic trafficking and metabolism to ensure its survival and proliferation (for review^3^). Infection-associated recruitment of immune cells leads to the formation of organized structures in the lungs, which may develop necrotic cores, known as granulomas, that can both restrict and sustain bacterial survival (for review^4^). Their rupture promotes bacterial dissemination and active disease (for review^5^). However, while granulomas are found in both active and latent TB infection (LTBI), their role in restricting bacillary growth and dissemination is considered a key contributor to the clinical state of latency. This form is clinically asymptomatic and affects an estimated 25% of the world’s population.^6^ As LTBI can persist for a lifetime, infected people are likely to be exposed to other infectious agents.

Here, we review recent studies on how TB modulates the host immune response during coinfection, highlighting the complexity and ongoing debate surrounding its impact on disease outcomes. In the first section, we examine how TB is a multifaceted disease with highly variable outcomes, as newly described disease states challenge the traditional binary classification of active and latent infection. This complexity is rarely considered in coinfection studies, complicating the interpretation of findings and limiting their translational relevance. This first section also introduces the key concepts of *Mtb* infection that frame the sections that follow. To also facilitate clarity, Table 1 summarizes the definitions of the terms used in this review concerning TB and coinfections. Following this, our initial focus is on past TB epidemics, where historical data and modeling provide insights into how ancient outbreaks shaped interactions with other pathogens. Subsequently, we turn to epidemiological data, which offer a glimpse into the global landscape of coinfections involving *Mtb* and their prevalence within populations. Finally, we explore translational and experimental models that connect epidemiological data with cellular mechanisms, shedding light on *Mtb*’s interactions with other pathogens at the immunological level. Through this analysis, we aim to enhance our understanding of host immunity in the context of coinfection with *Mtb*, and highlight the need for more integrative and multidisciplinary approaches to fully capture the complexity of coinfections with *Mtb*.

**Table 1:** Glossary of key terms related to tuberculosis and coinfections

Terminology	Definition (as used in this review)
TB (disease)	(tuberculosis) clinical disease caused by *Mycobacterium tuberculosis*, classically identified by immunological tests (e.g., TST or IGRA). In this review, the term “TB” is used to refer to tuberculosis disease in a general sense, encompassing the full spectrum of infection from latent to active states.
*Mtb*	(*Mycobacterium tuberculosis*) the bacterial pathogen responsible for tuberculosis, encompassing genetically diverse lineages with distinct epidemiological and immunological characteristics.
*Mtb* infection	(*Mycobacterium tuberculosis* infection) the presence of *Mycobacterium tuberculosis* within the host, regardless of clinical manifestation. The term “*Mtb* infection” is the most widely used throughout this review, particularly in *in vivo* and *in vitro* studies.
Active TB	(active tuberculosis) a state of *Mycobacterium tuberculosis* infection in which ongoing bacterial replication leads to clinical disease, with detectable pathology and transmissibility, accompanied by symptomatic, radiological, and/or microbiological evidence. In this review, the term “active TB” is used only when explicitly employed by the original study, with the corresponding detection method indicated in Tables S1 and S2.
LTBI	(latent tuberculosis infection) a state of persistent *Mycobacterium tuberculosis* infection without clinical signs or symptoms of disease, in which bacterial replication is contained by the host immune response, classically identified by immunological tests (e.g., TST or IGRA). In this review, the term “LTBI” is used only when explicitly employed by the original study, with the corresponding detection method indicated in Tables S1 and S2.
Coinfection	the simultaneous or sequential presence of two or more infectious agents within the same host, irrespective of whether these infections interact biologically or clinically. In this review, the term “coinfection” is also used when the temporal sequence of infections is unknown (when it is not clear which pathogen was acquired first).
Superinfection	acquisition of a secondary infectious agent occurring after the establishment of a primary infection. In this review, the term “superinfection” emphasizes the temporal sequence and is used to refer to the secondary infection.

This table provides definitions of key terms used throughout this review related to TB and coinfections. Given the heterogeneity of TB disease states and coinfection contexts, these definitions are intended to clarify terminology and improve consistency across sections.

## Beyond the duality: the spectrum between active and latent tuberculosis

### The spectrum of TB

TB is a complex disease that exists along a dynamic spectrum, ranging from complete bacterial clearance to active, symptomatic disease (for review^7^). Among those outcomes, recent research increasingly points to a wide diversity of TB states that are often challenging to distinguish from one another.

#### From clinical diversity to diagnostic challenges

Following exposure to *Mtb*, two broad outcomes are possible: elimination of the pathogen by the immune system or persistence of infection. Elimination of *Mtb* can occur either through innate immune response, leading to a negative tuberculin skin test (TST) or interferon-gamma release assay (IGRA), or through adaptive immune response,^8^ where TST and IGRA outcomes depend on memory T cell activation.^7^ Some individuals, called “resisters,” appear to be resistant to *Mtb* infection and remain TST/IGRA negative despite multiple exposures.^9^ Recent studies have shown that they develop *Mtb*-specific adaptive immune responses lacking IFN production, with CD4^+^ T cells displaying Th17- and regulatory-like features.^10^ Additionally, individuals called “reverters” show transient TST or IGRA positivity before reverting to test negativity over time, possibly indicating self-clearance of infection.^11^^,^^12^

If not cleared, *Mtb* persists in the body, mostly in the lungs but also in other tissues such as the bone marrow^13^ and adipose tissue.^14^ Most people control but do not eliminate the infection, leading to LTBI. In clinical and epidemiological studies, LTBI is generally inferred from a positive TST or IGRA in the absence of clinical evidence of active disease; however, these tests detect immune sensitization to Mtb antigens and cannot distinguish LTBI from other states within the TB spectrum.^15^ Within two years after infection, 5 to 15% of people will spontaneously progress to the active, symptomatic, and transmissible state, termed primary TB, while others remain at risk of reactivation, with an estimated 10% lifetime risk.^16^ Active TB causes cough, fever and weight loss, and is diagnosed via sputum smear, culture or molecular tests, as immune suppression or disease-induced anergy can cause false-negative TST/IGRA results.^17^ Additionally, some patients exhibit radiological evidence of TB and culture-positive disease without reporting symptoms, a condition referred to as subclinical TB.^18^ This form is increasingly recognized as an important contributor to transmission, despite typically lower bacterial loads, and may account for a large proportion of undiagnosed and untreated cases.^19^

Given the diverse manifestations of *Mtb* infection, distinguishing between disease states is critical for both clinical management and research. However, current diagnostic tools face significant limitations in accurately defining infection and disease state, making it difficult to differentiate latent infection from active disease or to confirm bacterial clearance. TST measures delayed-type hypersensitivity to mycobacterial antigens but cross-reacts with non-tuberculous mycobacteria and the current vaccinal strain *Mycobacterium bovis* Bacillus Calmette-Guérin (BCG), and therefore cannot differentiate latent from active TB.^20^ IGRA, introduced in the 2000s, quantifies IFN-γ responses to *Mtb*-specific antigens and avoids BCG interference, yet likewise fails to distinguish infection states. The diagnostic work-up for active TB has traditionally relied on chest radiography alongside bacteriological testing by smear microscopy and culture,^7^ while World Health Organization (WHO)-endorsed rapid molecular tests have increasingly become part of standardized diagnostic algorithms in recent years.^21^ Since 2014, WHO has been recommending LTBI screening in selected high-risk groups^22^ (people living with HIV, close contact with patients with active pulmonary TB, individuals at increased risk of progression due to immunosuppressive conditions or therapies). Altogether, diagnostic challenges persist across the TB spectrum, particularly for non-active and early disease states. Coinfections can further obscure the clinical picture by influencing the progression of TB.

#### Geographic distribution in TB manifestations

The complexity of TB and its diverse manifestations makes it challenging to fully grasp the global impact of the disease. TB burden is unevenly distributed across regions, and the majority of cases occur in low- and middle-income countries, where poverty, malnutrition, and limited healthcare access contribute to higher transmission rates.^7^ In 2023, most declared TB cases were in the WHO regions of South-East Asia (45%), Africa (24%) and the Western Pacific (17%), with lower shares in the Eastern Mediterranean (8.6%), the Americas (3.2%) and Europe (2.1%).^23^ This geographic disparity is also reflected in LTBI, which affects an estimated quarter of the global population. 80% of cases are located in South-East Asia (31%), the Pacific region (28%) and Africa (22%), while Europe accounts for 14% of cases.^24^ However, as mentioned before, only a small proportion of them will suffer from active disease during their lifetime. The large majority will never be diagnosed or experience symptoms, but many of them will encounter other pathogens throughout their life. It is therefore crucial to understand how LTBI affects the outcome of secondary infections, and conversely, whether these infections can impact the control of *Mtb*. Considering geographical context is essential, as regional disparities in global health, immune status, circulating *Mtb* lineages, and exposure to distinct coinfections critically shape patient status and must therefore be considered a unifying framework throughout the subsequent sections.

### Immune factors in pulmonary TB control

*Mtb* infection is characterized by complex and finely balanced immune mechanisms. This section briefly summarizes these key concepts to support the interpretation of the coinfection scenarios discussed in Section “bridging scales: from population patterns to animal models and cellular mechanisms of coinfection”. After inhaling infectious aerosols, *Mtb* reaches the pulmonary alveoli.^25^ There, pathogen-associated molecular patterns on the bacterial surface are sensed by host immune cells through pattern-recognition receptors, triggering phagocytosis primarily by macrophages, but also by dendritic cells and neutrophils.^26^^,^^27^ Toll-like receptors (TLRs) contribute to bacterial sensing and to the initiation of inflammatory and adaptive immune responses. Among them, TLR2 contributes to T cell co-stimulation by activating antigen-presenting cells, thereby promoting the production of IFN-γ and TNF-α.^28^ Similarly, TLR9 recognizes unmethylated CpG motifs in bacterial DNA, enhancing the activation of dendritic cells and promoting a Th1-biased immune response that is critical for controlling *Mtb* infection.^29^

These early sensing events not only initiate antimicrobial responses but also shape the recruitment and spatial organization of immune cells in the lung, ultimately leading to granuloma formation. In pulmonary TB, these structured aggregates of immune cells form in the lungs and represent a central feature of the host response to *Mtb*.^4^ They reflect the duality of *Mtb* infection: while granulomas can help contain the bacterium and prevent dissemination, they can also provide niches for bacterial persistence or replication.

Effective control of *Mtb* and prevention of reactivation depend on a balanced, coordinated immune response involving both innate and adaptive immunity. Macrophages are central to this control, activated primarily by IFN-γ to produce reactive nitrogen and oxygen intermediates and other antimicrobial mechanisms that limit bacterial replication.^30^^,^^31^ IFN-γ also induces chemokines like CXCL9 and CXCL10 that participate in adaptive cell recruitment to the granuloma site.^32^ TNF-α complements this by promoting macrophage activation and maintaining granuloma integrity,^33^ while it can promote CCL2 production. This chemokine contributes to monocyte recruitment to the site of infection, coordinating the cellular architecture of the granuloma.^34^ However, elevated levels of CCL2 have been observed in severe forms of TB, reflecting a more intense immune response associated with disease severity.^35^

In addition to macrophages, several innate immune cell populations, including dendritic cells,^36^ natural killer (NK) cells^37^ and neutrophils,^38^ contribute to early containment, organization of granulomas, as well as antigen presentation that primes the adaptive response.^39^ The role of eosinophils in TB is still poorly described, although *Mtb*-induced eosinophil recruitment has been shown to contribute in some settings beneficially to infection control.^40^

CD4^+^ T cells play an essential role in sustaining latency, not only through IFN-γ secretion but also by orchestrating immune interactions within the granuloma. Among these, Th1 cells drive macrophage activation, while Th17 cells contribute to mucosal immunity and granuloma structure through IL-17 signaling.^41^ In murine models, the absence of CD4^+^ T cells can lead to a loss of infection control, even when CD8^+^ T cells produce compensatory IFN-γ, suggesting additional regulatory and structural roles for CD4^+^ T cells.^42^ CD8^+^ T cells and unconventional T cells, such as γδ T cells and MAIT cells, also contribute to *Mtb* control through cytotoxic activity and rapid cytokine production, reinforcing the early containment of infection (for review, see^43^). Although IFN-γ and TNF-α levels are often elevated in active TB due to increased antigenic load,^44^ their sustained yet tightly regulated production is essential during latency.^45^ On the other hand, the anti-inflammatory cytokine IL-10 can suppress protective immunity by inhibiting macrophage activation, phagosome maturation, and Th1 responses.^46^ Dysregulation, whether through cytokine depletion, excessive inflammation or alternatively activated immune cells, can also lead to immunopathology or reactivation.^47^ Overall, maintaining TB control requires the sustained coordination of both innate and adaptive immune mechanisms to balance bacterial suppression with tissue preservation. In this context, the inflammatory environment induced by TB can influence the outcome of a secondary infection, while conversely, a secondary pathogen may disrupt the immune equilibrium required to contain the mycobacterial burden. The roles of the immune cell populations involved in TB, together with their key activating cytokines and secreted mediators, are detailed in Table S1.

### Modeling TB heterogeneity

While human studies are the most relevant for advancing TB research, they are also the most challenging. The interpretation of results is influenced by a variety of factors, including exposure duration and frequency, bacterial strain, inoculum size, disease severity, and the presence of comorbidities.^48^ Importantly, as discussed in Subsection “the spectrum of TB”, patients are often classified using imperfect or indirect criteria, making it challenging to accurately position individuals along the TB disease spectrum. This diagnostic uncertainty further complicates the interpretation and comparison of human studies across cohorts and settings.

Murine models are widely used due to their practicality, but they do not fully replicate human TB pathology, lacking features such as fully organized granulomas, caseous necrosis, and pronounced hypoxia.^49^ Chronic infection in mice has been employed to model LTBI, though the often higher and more sustained bacterial burden does not accurately reflect human latency.^50^ Nonetheless, these models are crucial for studying the immunological mechanisms and TB progression. Older studies suggest that during this persistent phase, the bacteria may enter a quiescent state, characterized by markedly reduced replication.^51^ Additionally, the chronic state that mice develop, sometimes induced by a low-dose infection protocol,^52^ has been utilized by several research groups as a model for LTBI.

Non-human primates, particularly macaques, better recapitulate the full spectrum of TB, including structured granulomas, but their use remains limited,^53^ especially in coinfection studies.

Other animal models, such as rabbits, guinea pigs, or zebrafish (often infected with *Mycobacterium marinum*), are also used in TB research (for review, see^54^), though not in the context of coinfection in the studies cited.

Overall, while animal models have significantly advanced our understanding of *Mtb* infection persistence and activation, human data still primarily rely on blood samples and clinical or imaging data, rather than direct lung analysis, making it difficult to fully capture the complexity of LTBI. When studying coinfections, it is crucial to develop methods to correlate findings from murine and non-human primate models with epidemiological data from human populations to bridge the gap between experimental and real-world outcomes.

In summary, this first section addresses a particularly challenging question, as TB is not a binary condition but spans a continuum of infection states that are difficult to define, diagnose, and compare across studies and populations. The position of an individual along the TB spectrum is shaped by a finely tuned and context-dependent immune equilibrium. This balance is influenced by numerous factors, including comorbidities, pathogen characteristics, and geographic and socio-epidemiological contexts. These layers of complexity not only complicate the interpretation of human data but also limit the translational relevance of experimental models. Under these conditions, understanding the bidirectional interactions between secondary infections and distinct TB states represents a major conceptual and methodological challenge, but also a critical step toward a more integrated view of TB pathogenesis and immune regulation.

## Echoes of the past: how ancient TB epidemics shaped the landscape of other infections

TB is among the most ancient infectious diseases, and it has been the focus of extensive medical research for many years.^55^ During the 20^th^ century, when TB remained widespread in Europe and North America, numerous studies examined its interactions with other infections, reflecting its high prevalence, chronic status and the frequent occurrence of coinfections.^56^^,^^57^^,^^58^^,^^59^ Two historical cases are worthy of particular note: the interaction between TB and influenza during the devastating 1889 and 1918 pandemics, and the hypothesis of cross-immunity between TB and leprosy.

### Mutual shaping of TB and influenza pandemics

Since the first influenza pandemic in 1580, 32 subsequent pandemics have been documented worldwide, with five major outbreaks in the 19^th^ and 20^th^ centuries.^60^ In 1922, Abbott hypothesized that the 1918 Spanish flu pandemic had accelerated the decline of TB through selective mortality; he suggested that *Mtb*-infected individuals were disproportionately killed by influenza, thereby reducing subsequent TB transmission and mortality.^56^ This hypothesis was later supported by demographic analyses, including the overlap between age patterns of influenza mortality and TB burden in the USA.^61^ Excess TB mortality during the influenza pandemic was also reported in Switzerland during the 1889 Russian influenza pandemic.^62^ Conversely, TB may have influenced the age-specific mortality pattern of influenza: in addition to the elderly and children, young adults showed elevated risk. Descriptive data from the USA, Japan, Switzerland and the Netherlands have revealed high influenza case fatality in this particular age group, hinting at hidden risk factors. Records from Swiss sanatoria confirmed that *Mtb*-infected patients had a higher risk of dying from influenza than non-TB controls. Finally, as previously noted, the sharp post-pandemic decline in TB deaths further supports TB’s role in shaping influenza mortality patterns.^63^ Interestingly, a study of two Norwegian sanatoriums revealed that *Mtb*-infected patients had lower rates of influenza morbidity compared to non-*Mtb*-infected staff members, likely due to reduced exposure and differential contact patterns. However, they experienced higher case fatality, especially young adult women.^64^ Collectively, historical and epidemiological data indicate a bidirectional interaction between TB and influenza. While influenza pandemics likely increased TB mortality through selective vulnerability of *Mtb*-infected individuals, TB itself appears to have shaped influenza mortality patterns, particularly among young adults. Importantly, lower influenza morbidity but higher case fatality among patients with TB suggest that exposure and immune status contribute differently to infection risk and disease outcome. Together, these observations highlight how chronic bacterial infections can modulate the impact of acute viral pandemics, an insight that remains highly relevant for pandemic preparedness.

### The TB/leprosy cross-immunity puzzle

**Figure 1:**
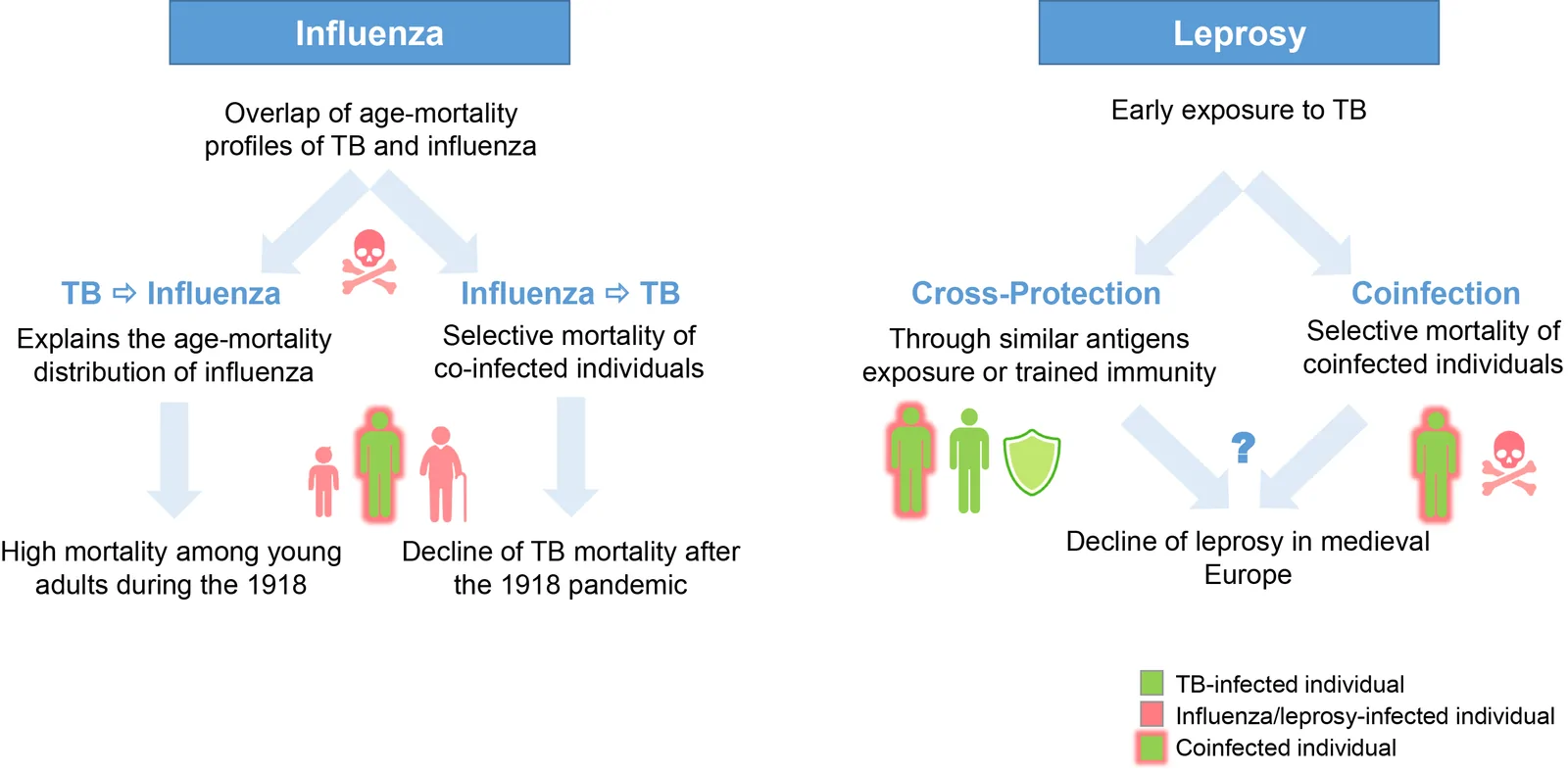
Historical TB/influenza and TB/leprosy coinfection models During the 1918 influenza pandemic, TB is thought to have shaped mortality curves by disproportionately affecting adults with underlying TB, unlike infants or the elderly. In contrast, in medieval Europe, the decline of leprosy may have resulted from cross-protection conferred by TB infection or from selective mortality among coinfected individuals. Icons from BioRender. Dagan, Y. (2026) (https://BioRender.com/y4mcdyo).

Leprosy was endemic in Medieval Europe but declined significantly beginning around the 14^th^ century,^65^ whereas TB persisted at epidemic levels, peaking during the so-called “White Plague” in the 17^th^ and 18^th^ centuries (for review^66^). Chaussinand was the first to hypothesize cross-immunity between TB and leprosy; based on animal experiments and epidemiological data, he theorized a disease antagonism explaining the decline of leprosy in areas where TB had become prevalent.^58^ Since then, several studies have investigated the interaction between TB and leprosy during the Middle Ages. Crespo *et al.* reviewed key parameters involved in potential cross-protection between *Mtb* and *Mycobacterium leprae*, the causative agent of leprosy, compiling historical and modern evidence in support of this idea.^67^ However, they also emphasized the complexity of this interaction, notably due to ecological and social factors that create heterogeneous epidemiological landscapes, making it difficult to isolate TB’s impact on leprosy’s decline.^68^^,^^69^ The clinical manifestations of both infections may also matter, as TB is thought to protect specifically against tuberculoid leprosy, the more prevalent form in late Medieval times, rather than the lepromatous form.^70^ Moreover, the historical prevalence of TB has likely been underestimated, given its latent nature and the frequent absence of bone lesions, used as the primary marker for diagnosing the infection in skeletal remains.^71^ Lietman *et al.* tested Chaussinand’s theory through transmission modeling and found that, given leprosy’s slow reproductive rate, TB could indeed have contributed to its disappearance over centuries.^72^ The alternative idea of selective mortality among coinfected individuals has also been considered, based on the immunological changes observed in archaeological samples containing DNA from both pathogens,^73^ and on the mathematical modeling of this coinfection hypothesis.^74^ Overall, these multidisciplinary findings suggest that the interaction between TB and leprosy cannot be explained by a single factor, but rather reflects the convergence of biological, clinical and socio-environmental determinants. While the possibility of *Mtb*-mediated protection marks a key conceptual turning point, this effect appears context-dependent, potentially restricted to specific clinical forms, and is further complicated by the likely underestimation of TB prevalence in historical records. This framework, summarized in Figure 1, provides a conceptual basis for exploring the immunological mechanisms underlying potential cross-protection, discussed in Section “bridging scales: from population patterns to animal models and cellular mechanisms of coinfection”.

The long-standing interest in coinfections with *Mtb*, illustrated by its historical interactions with leprosy and influenza, highlights the relevance of revisiting this question in modern contexts. Together, these examples suggest that TB may have acted as a major regulator of past epidemics by shaping host susceptibility, disease severity, and transmission potential of concurrent infections, thereby influencing the overall dynamics of secondary pathogens.

In the following sections of the review, epidemiological and experimental studies are presented separately to provide a clearer understanding of the evidence from each approach. Within both sections, coinfections are categorized according to their reported outcome: exacerbating, neutral, or protective against one or the other infection. Table S2 provides an overview of all pathogens studied in combination with TB, including their host, target organs, modes of transmission, and associated diseases.

## Epidemiological clues: tracing the impact of *Mtb* on human coinfections

Areas with high TB incidence frequently overlap with other infectious diseases in endemic regions, yet coinfections remain understudied. This is especially relevant in high-burden countries, such as India, Indonesia, China, the Philippines, Pakistan, Nigeria, Bangladesh, and the Democratic Republic of the Congo, which together account for over two-thirds of global TB cases.^23^ In these environments, populations are also affected by HIV, malaria, helminths, enteric pathogens, and opportunistic bacterial and fungal pathogens.^75^ These infections can alter TB pathogenesis, diagnosis, and treatment outcomes. However, current data on coinfections are fragmented, often limited to single pathogens and specific localities. The urgency to adopt a more integrated research approach is heightened by global shifts such as climate change, urbanization, and increased human mobility, which facilitate the emergence and spread of infectious diseases.^76^

In this review, we have compiled sixty-six cohort and analytical studies addressing coinfections with *Mtb*, summarized in Table S3. This descriptive table gathers all publications discussed in this section, providing the foundation for the comparative discussion that follows. Moreover, given the frequent lack of precision in defining TB states, we used only the terminology explicitly provided by each study: we refer to LTBI when specified, and otherwise use the term *Mtb* infection. Building on this, we aim to understand the real-world landscape of *Mtb* coinfections in humans, including their distribution, impact on TB outcomes, and potential contributions to increased case numbers or disease severity.

### The synergistic threat of *Mtb*/HIV coinfection

*Mtb* and HIV coinfection is widely recognized as the most significant infectious risk factor for TB progression. In 2023, approximately 24% of the 660,000 individuals with TB and HIV died compared to 11% of those without HIV who died from TB.^77^ TB remains the leading cause of hospitalization among HIV-infected adults (18%) and children (10%), and TB-related in-hospital mortality worldwide was 25% among adults and 30% among children with HIV infection.^78^ HIV increases risk of TB progression to active disease, primarily by disrupting local immune responses within granulomas.^79^ Notably, people living with chronic HIV infection show significantly reduced *Mtb*-specific Th1 cells.^80^ This disruption can double the incidence of TB within the first year of HIV infection^81^^,^^82^ and increases the risk of LTBI reactivation.^83^^,^^84^ Conversely, active TB may enhance HIV-1 replication, promoting early viral dissemination.^85^ Both latent and active TB have been associated with strong immune activation in patient samples, which can accelerate HIV progression.^86^^,^^87^ HIV also predisposes individuals to further opportunistic infections. Notably, coinfection between *Mtb* and *Pneumocystis carinii* has been linked to more severe pneumonia in HIV-infected patients,^88^ and *Mtb*/*Streptococcus pneumoniae* coinfections have been documented in HIV-positive individuals.^89^^,^^90^ *Mtb* infection is also a suspected factor in exacerbation in HIV/*Leishmania donovani* coinfection.^91^

While most studies underscore the exacerbating nature of TB/HIV coinfection, recent findings suggest that asymptomatic TB may confer some degree of immune modulation: Kusejko *et al.* reported lower HIV viral loads in individuals with LTBI compared to TB-negative patients.^92^ Additionally, Tepekule *et al.* showed that transcriptomic changes in peripheral blood mononuclear cells (PBMCs) from HIV-positive individuals with LTBI were associated with baseline immune activation and reduced viremia, a phenomenon that was absent in active TB.^93^ These results suggest a complex interplay between TB status and HIV progression, raising the possibility that asymptomatic *Mtb* infection may confer a degree of heterologous protection. However, these findings remain preliminary and are derived from limited cohorts; further investigation is needed to assess their robustness and generalizability. Together, these data highlight the need for nuanced and context-specific studies of *Mtb*/HIV coinfection, as well as dedicated *in vitro* investigations to elucidate the underlying immunological mechanisms; these topics will be explored in more detail later in Section “bridging scales: from population patterns to animal models and cellular mechanisms of coinfection” of this review.

### Increased susceptibility and severity in coinfections with *Mtb*

In this section, we examine how TB infection may alter susceptibility to, or the clinical course of, other infectious diseases. Because TB and other infections are associated with immune perturbations, coinfections are often reported as increased susceptibility and/or exacerbated outcomes, an observation that also applies to TB. In addition to HIV, other chronic viral infections, such as cytomegalovirus (CMV) and hepatitis B and C virus (HBV and HCV), are established TB risk factors. CMV seropositivity is linked to LTBI^94^ and increased progression to active TB,^95^^,^^96^ particularly in children infected with CMV within their first year.^97^ With an overall prevalence of 7% among *Mtb*-infected patients,^98^ HCV infection is associated with higher TB disease risk.^99^^,^^100^ In a UK cohort, 18.4% of patients with newly diagnosed TB had HBV or HCV markers, higher than the national average, with coinfection prevalence greater in active TB compared to LTBI.^101^ Nonetheless, rates of coinfection among individuals with LTBI remain elevated, as also observed in Southern California between 2008 and 2019.^102^

Regarding acute viral infections, influenza viruses provide a notable model for studying the impact on *Mtb* infection. Epidemiological data often show an increase in influenza incidence among patients with TB^103^^,^^104^ or a worsening of either disease during coinfection,^105^^,^^106^^,^^107^ which is consistent with the historical data described in Subsection “mutual shaping of TB and influenza pandemics”. However, many studies also refrain from drawing a clear conclusion, instead suggesting a neutral or inconclusive interaction between the two diseases.^108^^,^^109^^,^^110^^,^^111^^,^^112^^,^^113^

Turning to bacterial coinfections, they are frequently observed in settings with a high TB burden. In Far East Russia, where TB incidence reached 105.7 per 100,000 in 2008, children with *Mtb* infection were more frequently colonized by *Haemophilus influenzae* than healthy controls, leading to acute upper and lower respiratory tract infections.^114^ Similarly, Gram-negative bacteria, such as *Klebsiella* spp. and *Pseudomonas* spp., were found in 33% of *Mtb*-infected patients’ sputum, compared to 9% of all patients, in a Cambodian cohort.^115^ These findings suggest that individuals with TB experience an increased burden and more severe pathological outcomes related to bacterial superinfections. A similar trend can be observed for some fungal infections, with a high prevalence of *Candida* spp. in *Mtb*-infected patients’ sputum.^116^^,^^117^^,^^118^

Finally, parasitic coinfections further illustrate the complexity of TB-associated susceptibility. In countries with high parasitic prevalence, parasitic species are found to be concurrent with *Mtb* infection in multiple organs, which increases antibacterial therapy intolerance and deteriorates TB disease prognosis.^119^ These patterns illustrate how parasitic coinfections can amplify disease severity rather than merely coexist. Despite the large geographical overlap between endemic regions, few studies document the cross-sectional prevalence of malaria in *Mtb*-infected patients, with rates ranging from 5% to 37% depending on the regions and parameters considered.^120^^,^^121^ However, an ecological study based on epidemiological surveillance data from the Brazilian Amazon revealed a spatial association between malaria and TB, independently of socio-economic factors, suggesting potential synergistic drivers of disease burden at the population level.^122^ Similarly, helminths and *Mtb* infections largely overlap at the population and geographic level, with a pooled prevalence of 29.69%.^123^ In Brazil and Ethiopia, active TB was significantly associated with intestinal helminth infections.^124^^,^^125^ However, in HIV-positive populations in Uganda and Brazil, no association was found with gastrointestinal parasites or *Mansonella perstans*,^126^ although schistosomiasis was linked to TB progression in Uganda.^127^ Taken together, these studies highlight the heterogeneous impact of parasitic coinfections on TB outcomes, with effects ranging from increased susceptibility and disease progression to the absence of detectable associations, underscoring the importance of pathogen-specific and context-dependent interactions.

In some of these studies, additional parameters challenge the causal link between TB and coinfections. For instance, the higher prevalence of Gram-negative bacteria observed in Cambodian *Mtb*-infected patients may be attributed to lung cavitation, which independently favors bacterial colonization.^115^ Likewise, factors such as living conditions, limited access to healthcare, and poor sanitation represent additional confounders that could bias the observed associations; however, these risk factors are rarely acknowledged and thoroughly investigated in epidemiological coinfection studies. The example of influenza also highlights that not all interactions follow a straightforward exacerbation pattern, with several studies reporting neutral or inconclusive outcomes.^103^^,^^104^^,^^105^^,^^106^^,^^107^^,^^108^^,^^109^^,^^110^^,^^111^^,^^112^^,^^113^

Overall, coinfections in the context of TB are frequently associated with increased susceptibility to, or greater severity of, secondary infections, as a wide range of pathogens appear more common among *Mtb*-infected individuals. However, establishing a causal link remains challenging, as in many cohorts, additional factors can independently facilitate colonization or symptomatic disease. Altogether, these variables highlight the challenge of distinguishing the direct immunological impact of *Mtb* infection from the secondary consequences of tissue damage and chronic inflammation, thereby underscoring the necessity for enhanced surveillance of patients.

### Hints of protection in *Mtb* coinfections

The potential protective effect of *Mtb* infection against other pathogens has recently emerged as a surprising and largely understudied phenomenon. In addition to the historical data presented in Subsection “the TB/leprosy cross-immunity puzzle”, the possible cross-protection between TB and leprosy has been shown in 2016 in the Marshallese population in Arkansas, USA, where a negative association was found between the presence of anti-*Mycobacterium leprae* antibodies and positive IGRA tests.^128^ Similar observations have been reported more unexpectedly in unrelated infectious contexts. In a large population-based study in Tennessee, the incidence of *Chlamydia trachomatis* infection was found to be reduced within the first year post-TB diagnosis.^129^ In line with these findings, Kusejko *et al.* also reported reduced incidence of opportunistic infections such as candidiasis and oral hairy leukoplakia in individuals with LTBI, compared to healthy individuals.^92^ However, these observations are largely associative and do not establish causality.

In contrast to the studies mentioned earlier, in a Tanzanian cohort, pathogenic respiratory bacteria were less likely to be detected in *Mtb*-infected patients than in controls.^130^ Similarly, a study in Ethiopia found that asymptomatic helminth infection correlated with lower sputum smear positivity in active TB, suggesting reduced *Mtb* bacterial burden.^131^ These contrasting observations raise the possibility of pathogen-pathogen interactions, either affecting susceptibility to secondary infections or modulating *Mtb* burden.

Elsewhere, Lee *et al.* observed a significant decrease in the incidence of TB among people infected with measles in the 2000–2001 outbreak in Korea compared to the general population, but they did not conclude that measles reduces the risk of TB as no plausible mechanisms have been elucidated.^132^ Other explanations, such as improved healthcare access, have been proposed for low coinfection rates of *Streptococcus pneumoniae* and *Mtb* in tertiary health facilities in Nigeria.^133^

Recently, the COVID-19 pandemic has offered unexpected insights into *Mtb*-related immune modulation. While some case reports initially raised concerns about the reactivation of LTBI following SARS-CoV-2 infection, these were often based on hospitalized patients and may have been biased toward symptomatic coinfections.^134^ At a population level, Inoue *et al.* demonstrated an inverse correlation between the burden of past TB epidemics, particularly those of the 1950s, and COVID-19 incidence and mortality.^135^ Supporting this idea, Takahashi employed instrumental variable analysis to show a significant association between LTBI prevalence and reduced COVID-19 mortality.^136^ However, large population-based studies like these remain rare, and current data are insufficient to determine whether *Mtb* exacerbates, protects from, or has no impact on the outcome of secondary infections.

### Interpretive limitations of current evidence

Despite these numerous reports suggesting either exacerbation or protection, most epidemiological studies available on coinfections with TB simply document their presence. Several studies have highlighted the occurrence of diverse bacterial coinfections, without drawing clear conclusions regarding the severity outcome.^137^^,^^138^^,^^139^^,^^140^ Among the coinfections discussed earlier in this review, studies on *Streptococcus pneumoniae*, *Aspergillus* spp. and *Candida* spp. report their presence in TB cohorts, but do not include comparisons with non-*Mtb*-infected populations.^141^^,^^142^^,^^143^^,^^144^^,^^145^ No association between the risk of infection with *Leishmania donovani* and *Mtb* coinfection was reported in a village in Eastern Sudan,^146^ and the same applies to non-tuberculous mycobacteria in a cohort in rural Mozambique^147^ or in Taiwan. For the latter, an increase in the prevalence has been shown between 2005 and 2013, but with no clear evaluation of the coinfection outcome.^148^ Moreover, we found no epidemiological studies on several prevalent past and present epidemics, for instance plague, syphilis and flavivirus infections.

Overall, these findings highlight the dual nature of TB coinfections: while many studies report the detrimental impact of coinfection on TB outcomes, a subset of epidemiological data suggest that *Mtb* infection may also exert protective effects in specific contexts. These findings in human data reflect the heterogeneity of host-pathogen interactions, as one pathogen may modulate the immune environment in ways that limit the other’s progression; mechanisms that are discussed further in Section “bridging scales: from population patterns to animal models and cellular mechanisms of coinfection” of this review. Importantly, the scarcity and inconsistency of such observations call for cautious interpretation, as a considerable number of studies find no clear association between *Mtb* infection and the coinfection outcome. Furthermore, epidemiological associations may be influenced by numerous confounding factors, including HIV status, malnutrition, diabetes, smoking, antimicrobial exposure, socioeconomic conditions, and access to healthcare, which are not always adequately accounted for across studies. This lack of consistent direction, combined with the broad spectrum of TB and its immunological manifestations, emphasizes the need for more systematic investigation, especially in TB-endemic regions, and the relevance of understanding the mechanisms behind these interactions.

## Bridging scales: from population patterns to animal models and cellular mechanisms of coinfection

Given the limited epidemiological data on coinfections involving *Mtb*, the scarcity of patient sample investigations and mechanistic studies exploring the underlying immune processes are not surprising. On one hand, clinical samples from coinfected individuals are rare and often not collected systematically, and identifying specific markers for either infection can be challenging. On the other hand, experimental coinfection models, whether *in vitro* or *in vivo*, present significant technical difficulties, as they require careful control and compatibility of both infectious agents and their respective model systems.

Despite these challenges, several studies have investigated the immunological mechanisms and implications of TB coinfection. We have gathered in Table S4 thirty-eight publications, including *in vivo*, *in vitro,* and *ex vivo* experiments, as well as studies based on patient-derived samples. Importantly, the choice of infection sequence and kinetics strongly shapes the research question: one may examine how a secondary infection affects *Mtb* control, or conversely, whether pre-existing TB alters the host’s ability to control a subsequent infection. The order of infection is indicated in the tables whenever this information was available. However, comparing infection sequences across different pathogens remains difficult due to the heterogeneity of study designs. Not all combinations have been experimentally investigated, and when they have, methodological differences often preclude meaningful comparisons.

This section on mechanistic investigations is conceptually divided into two complementary approaches: one examines how secondary infections influence the control of TB, while the other investigates whether pre-existing *Mtb* infection modulates the host’s ability to respond to subsequent infections.

### Coinfection-driven imbalance of TB immune control

Coinfections represent a critical but often underestimated factor influencing the trajectory of *Mtb* infection. By altering the balance between protective and regulatory immune responses, concurrent pathogens can drive TB pathogenesis toward exacerbation, but intriguingly, under certain circumstances, they may also confer unexpected protection. Understanding these dual outcomes is essential to unravel the complex interplay between *Mtb* and the host immune system, as well as to identify common immunological mechanisms underlying susceptibility or resilience. To improve clarity, the following sections are therefore organized not by the nature of the secondary pathogen, but by the type of immune response that disrupts TB control.

#### Innate response alteration

Parasitic coinfections provide a clear illustration of how coinfecting organisms can either impair or enhance *Mtb* control through modulation of innate immunity. For instance, filarial antigens disrupt antigen-presenting cell maturation and pro-inflammatory cytokine expression in response to *Mtb*.^149^ Consistently, filarial infections induce the downregulation of TLR2 and TLR9 expression and function,^150^ which are involved in Th1 responses.^29^ However, parasitic infections do not uniformly impair immunity; in some cases, they appear to enhance early control of *Mtb*. Hookworm-infected patients’ blood had a significantly greater ability to limit *Mtb* growth *in vitro*, which was lost following hookworm treatment; this enhanced control is accompanied by a significant negative relationship between mycobacterial growth and eosinophil counts.^151^ Moreover, human phagocytes had an increased ability to control *Mtb* during early stages of helminth exposure. Pre-exposure of monocytes or macrophages, respectively collected from human PBMCs and differentiated from PBMCs, to *Ascaris lumbricoides* or *Schistosoma mansoni* proteins increased their ability to control intracellular *Mtb* growth; in these settings, the effects were direct and did not involve T cells.^152^ Yet, helminth infections have also been shown to impair *Mtb* control by promoting accumulation of alternatively activated macrophages through IL-4 signaling.^153^ Similarly, using *Plasmodium falciparum*-infected erythrocytes in a co-culture model, Hawkes *et al.* demonstrated that superinfection with this protozoan impairs the control of *Mtb* replication in macrophages. The authors also showed that *Plasmodium falciparum*-infected mice experienced an exacerbation of TB disease caused by the disruption of granulomatous lesions and the influx of dysfunctional monocytes.^154^ Similar results were reported in the setting of *Mtb*/*Plasmodium yoelii* coinfection.^155^ These observations indicate that parasitic coinfections converge on a limited set of shared immunological mechanisms, such as modulation of cytokine signaling, cell polarization, and granuloma integrity, that can either impair or transiently enhance control of *Mtb*. This mechanistic diversity reinforces the need to interpret individual studies within these broader immunological themes.

#### Adaptive response dysfunction

Adaptive immune dysfunction underlies many of the deleterious effects of coinfection on *Mtb* control. HIV is a well-documented example of how coinfection disrupts *Mtb* control, as it profoundly depletes CD4^+^ T cells, which are essential for controlling *Mtb*. In cynomolgus macaques, reactivation of LTBI following simian immunodeficiency virus (SIV) infection correlates with early T cell loss.^156^ HIV-1 and SIV infections are shown to deplete lung interstitial CD4^+^ T cells, promoting *Mtb* dissemination through peripheral organs.^157^ Yet, Bucşan *et al.* showed that CD4^+^ T cell depletion alone is insufficient to drive reactivation, as macaques treated with CD4-depleting antibodies did not consistently develop active TB, in contrast to SIV-infected animals, suggesting that additional virus-induced factors lead to reactivation.^158^ Influenza A virus (IAV) illustrates how acute viral coinfections can also disrupt adaptive immune responses. Coinfection with IAV alters the overall inflammatory profile during TB, characterized by lower levels of IL-17A in the sputum, higher CCL2 levels in the blood, as well as increased frequencies of IFN-γ^+^IL-17^+^CD4^+^ and IFN-γ^+^CD8^+^ T cells, which are dysregulated T cell populations, both associated with poor TB control.^159^ Consistently, pulmonary coinfection with IAV causes an impairment of the generation of effective CD8^+^ and CD4^+^ T cell responses capable of controlling concurrent mycobacterial infection.^160^ Together, these changes upon HIV or IAV infection reflect the detrimental impact of a dysregulated immune environment on the control of *Mtb* infection. Other pathogens can similarly perturb adaptive immunity, highlighting a convergent mechanism of TB exacerbation. Among children with TB, CMV is associated with a lower frequency of CD3^−^ CD4^−^ CD8^−^ lymphocytes and lower frequency of NK cells in the blood.^161^ Chronic *Helicobacter hepaticus* colonization in mice induces gut dysbiosis and systemic inflammation that leads to a marked accumulation of activated pulmonary CD4^+^ and CD8^+^ T cells following *Mtb* challenge, associated with excessive cytokine production, loss of immune control, and severe lung tissue damage.^162^ Together, these studies illustrate that coinfections can compromise TB control through dysfunction of adaptive immunity, including T cell depletion, skewed cytokine responses, and impaired effector function. By highlighting these shared mechanisms, they underscore how diverse pathogens converge on the adaptive arm of the immune system to exacerbate TB.

#### Interferon-mediated response

Interferons are central cytokines orchestrating immune responses against intracellular pathogens. Th1 CD4^+^ T cells are the main source of IFN-γ, the essential cytokine for macrophage activation and bacterial control. The course of TB during coinfection largely depends on the impact of the secondary pathogen on these key components. Protective modulation of IFN-γ responses has been observed. For instance, in humans and non-human primates, *Helicobacter pylori* infection is associated with protection against *Mtb* through enhanced IFN-γ and Th1-like responses.^163^ Conversely, many coinfections impair antigen-specific cellular responses; for this reason, IFN-γ-mediated immunity is not uniformly protective and strongly depends on its context and specificity. For example, among children with TB, CMV-specific IFN-γ response positively correlates with increased disease risk.^161^ Conversely, influenza infection suppresses *Mtb*-specific IFN-γ responses through type I IFN signaling, leading to impaired pulmonary recruitment of protective CD4^+^ Th1 cells and promoting hyperinflammation and *Mtb* growth.^159^^,^^160^^,^^164^ Helminth infections illustrate the complex duality of these effects. Hookworm infections shift the immune response from protective Th1/Th17 (IFN-γ, TNF-α, IL-17A) toward regulatory T cells and Th2 (IL-10, TGFβ, IL-4, IL-13).^165^^,^^166^ In patients with active TB, helminths reduce IFN-γ and dual Th1/Th17 responses, which are beneficial for *Mtb* control, while increasing IL-10, Th2 and regulatory T cells activities.^167^^,^^168^ In LTBI, filarial infection also reduces Th1 and Th17 responses through upregulation of checkpoint molecules such as CTLA-4 and PD-1^169^; they also induce the expansion of CD4^+^IL-4^+^ memory T cells, likely suppressing Th1 cell development via Th1/Th2 cross-regulation.^170^ Together, these studies demonstrate that IFN-γ responses are a central target of coinfection-driven immune modulation. However, their impact on TB outcome is highly context-dependent, as protection is determined not only by the magnitude of IFN-γ production but also by its cellular source, antigen specificity, and the broader immune environment in which it is generated.

Type I IFNs are essential antiviral mediators, promoting viral clearance, activating dendritic cells, and enhancing CD8^+^ T cell responses. However, in the context of bacterial or chronic viral infections, type I IFNs can paradoxically have detrimental effects. In the context of TB, type I IFN signaling has been consistently associated with exacerbated disease progression and impaired bacterial control.^171^ This has been demonstrated in murine coinfection models: Redford *et al.* showed that prior infection with IAV worsened subsequent *Mtb* infection through a type I IFN-dependent mechanism, leading to increased mycobacterial burden.^160^ Similarly, infection with lymphocytic choriomeningitis virus (LCMV), a chronic viral infection, amplifies TB severity in mice by promoting type I IFN-driven immune dysregulation, including the suppression of *Mtb*-specific IFN-γ production and CXCL9/10 chemokine expression.^172^ Human studies support this dual role of type I IFNs: CMV-induced T cell activation and type I IFN production could increase the risk of TB disease severity.^173^ Collectively, these studies highlight the dual nature of type I IFN signaling: while critical for antiviral defense, sustained or excessive type I IFN responses during coinfection can disrupt protective antimycobacterial immunity and contribute to TB pathogenesis.

Together, these studies illustrate that interferon-mediated pathways are a major target of coinfection-driven immune modulation. Coinfecting pathogens can either impair or enhance IFN-γ responses, and type I IFN signaling often acts as a double-edged sword, promoting antiviral immunity while potentially undermining control of *Mtb*. This mechanistic convergence highlights the central role of interferon signaling in determining the outcome of TB during coinfection.

#### Immunomodulatory shifts

During coinfections, the host immune response to *Mtb* is shaped by complex immunomodulatory shifts, in which both pro- and anti-inflammatory pathways may contribute to protection or disease progression depending on the context.

TNF-α plays an important role in the maintenance of LTBI and long-term control of *Mtb* infection.^54^ In an LCMV/*Mtb* murine coinfection model, the increased amount of TNF-α compared to *Mtb* infection alone initially impaired *Mtb* growth, but the delayed lymph node transport and the antigen-specific T cell priming ultimately allow the immunologically concealed expansion of *Mtb*, in the absence of effective early immunosurveillance,^174^ which highlights that TNF-α responses can both support protection and, if dysregulated, contribute to TB progression during coinfection, underscoring the importance of time-dependent regulation of this cytokine in controlling *Mtb*.

Conversely, the anti-inflammatory cytokine IL-10 is a critical immunoregulatory cytokine in *Mtb* infection and plays a key role in susceptibility, as it was previously described to worsen TB infection by preventing phagosome maturation and phagolysosomal fusion.^175^ Across multiple coinfection settings, dysregulated IL-10 responses emerge as a recurrent mechanism associated with increased susceptibility or exacerbation of TB. Several pathogens have been shown to directly or indirectly amplify IL-10-mediated immunosuppression in the context of *Mtb* infection. CMV produces a functional analog to human IL-10, possibly worsening TB infection by reinforcing anti-inflammatory signaling.^176^ In malaria/TB coinfected patients, IL-10 production is increased compared to mono-infected groups, thereby promoting an anti-inflammatory response against *Mtb*, whereas IL-10 levels remain significantly lower in patients with LTBI.^177^ Similarly, it has been shown that intestinal helminth coinfection is accompanied by an increased production of IL-10 in individuals with active pulmonary TB.^167^ Experimental evidence further supports a causal role for this pathway, as blocking the IL-10 receptor in IAV/*Mtb* coinfected mice reduces bacterial burden to levels observed in *Mtb*-only infections, highlighting a potential therapeutic target.^164^ These findings illustrate that dysregulated IL-10 signaling can undermine TB immunity, and that modulation of this pathway represents a convergent mechanism by which coinfections influence TB outcomes.

Overall, insufficient pro-inflammatory and excessive anti-inflammatory responses can compromise TB control, highlighting the importance of tightly regulated cytokine signaling during coinfection.

**Figure 2:**
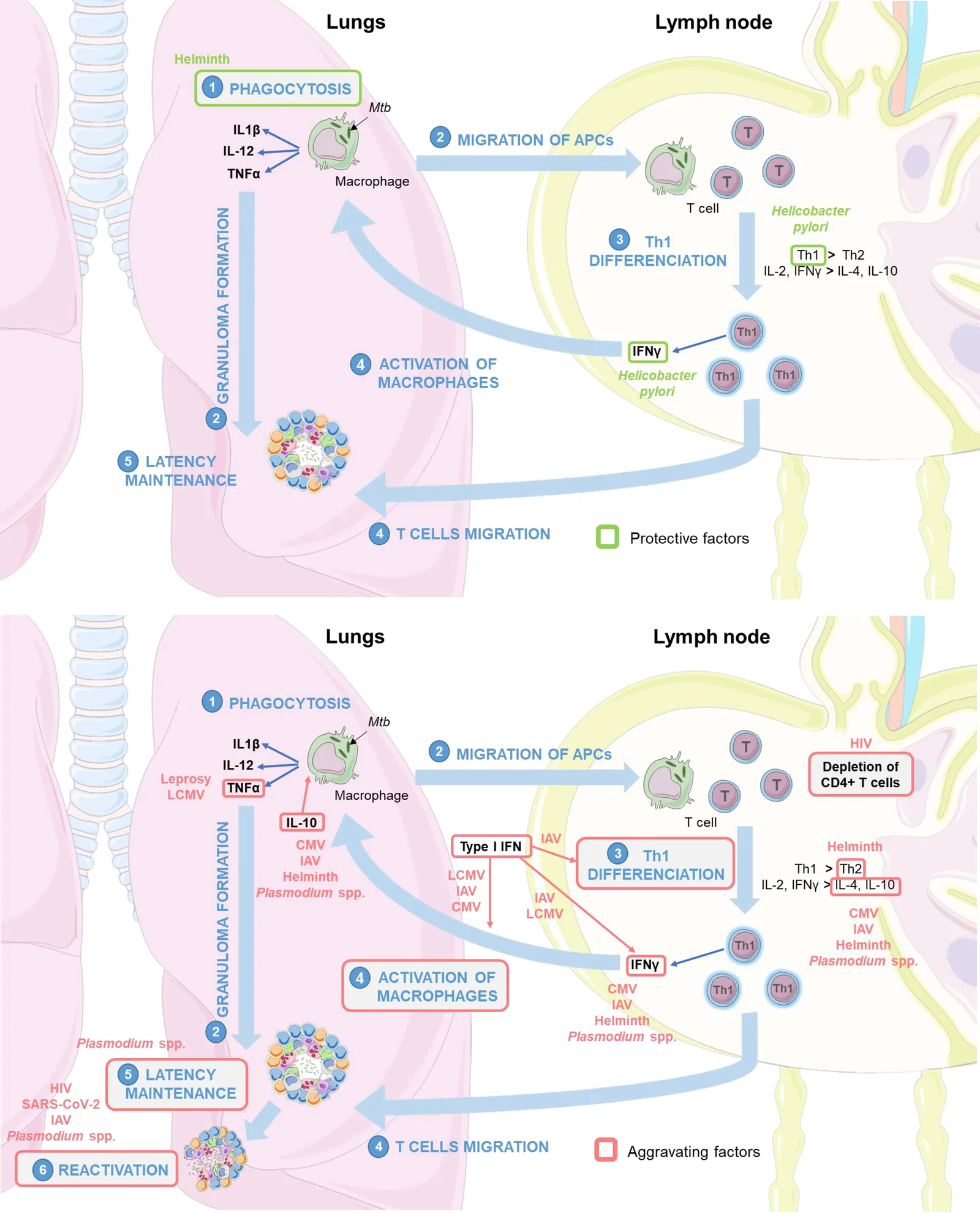
Possible modulatory effects of coinfections on TB progression Schematic representation of the cellular interplay during *Mtb* infection, with a focus on macrophage and T cell interactions that contribute to granuloma formation and containment of the bacilli. The figure also highlights how coinfecting pathogens, through modulation of cytokine production, macrophage activation, or T cell functionality, can alter the local immune environment and either impair or enhance the host’s ability to control TB progression. The upper part of the figure illustrates immune mechanisms and coinfection-induced factors associated with protection against TB (green symbols), whereas the lower part depicts mechanisms that exacerbate disease progression (red symbols). LCMV: lymphocytic choriomeningitis virus; IAV: influenza A virus; CMV: cytomegalovirus; HIV: human immunodeficiency virus. Steps 2 and 4 are intentionally repeated across the figure to illustrate processes that occur simultaneously rather than sequentially. The figure was partly created using Servier Medical Art templates, provided by Servier, which are licensed under a Creative Commons Attribution 4.0 Unported License (http://smart.servier.com).

Concurrent infections can profoundly influence the outcome of TB, either exacerbating disease progression or conferring partial protection. These opposing effects are mediated through a variety of immune mechanisms, including modulation of cytokine environments, alteration of antigen presentation, and competition for immune resources. Both innate and adaptive responses are affected, shaping the host’s ability to contain or eliminate *Mtb*. These mechanisms, and their consequences on TB control, are summarized in Figure 2. Importantly, studying the impact of these coinfections on *Mtb* infection provides dual insights: on one hand, it helps elucidate the immunological processes underlying TB reactivation or loss of bacterial control, identifying key pathways that predispose to disease progression; on the other hand, it can reveal novel targets for therapeutic intervention, as interventions aimed at modulating cytokine imbalances, checkpoint pathways, or interferon responses could restore protective immunity or limit pathological inflammation.

### TB-primed immunity against coinfections

While much attention has been given to how coinfections influence TB progression, less is known about how the disease itself shapes the host response to secondary infections. Understanding this directionality is particularly important, as *Mtb*-induced immune remodeling, particularly in the case of undiagnosed LTBI, may alter susceptibility to, or the severity of, subsequent infections. However, *in vitro* studies addressing this question remain scarce, largely due to the limited availability of experimental models that faithfully recapitulate LTBI.

#### The Mackaness effect and bacterial coinfections

This possible protection caused by *Mtb* is not a new concept. A 1964 study, which led to what is now known as the “Mackaness effect,” described the existence of cross-protection in mice infected with three different species of intracellular bacteria: *Listeria monocytogenes*, *Brucella abortus* and *Mtb*.^57^ Subsequent studies have also demonstrated that infection of mice with *Mtb* conferred protection against *Listeria monocytogenes*.^59^ Nemeth *et al.* showed that a contained *Mtb* infection induces heterologous protection in mice as it reduces the bacterial burden of *Listeria monocytogenes* and the number of metastases caused by the B16-F10 melanoma cell line.^178^ This protection was associated with an enhanced activation of alveolar macrophages and accelerated recruitment of *Mtb*-specific T cells to the lung parenchyma. Moreover, the protection was also enhanced upon re-exposure in macaques to *Mtb* through aerosol challenge.^179^ Together, these findings suggest that *Mtb* infection can induce a broad state of heterologous protection against distinct intracellular pathogens, supporting the concept that chronic *Mtb* infection may reprogram innate and adaptive immune responses beyond TB-specific immunity.

#### TB-driven resistance against respiratory viruses

In the following section, SARS-CoV-2 serves as a primary model of acute respiratory viral infection, and given the large number of studies on *Mtb*/SARS-CoV-2 coinfection, it provides a framework to investigate how pre-existing *Mtb* infection influences antiviral immunity. In this context, several experimental studies describe a beneficial effect of chronic *Mtb* infection on SARS-CoV-2.

Evidence from mouse models demonstrates that pre-infection with *Mtb* reduces SARS-CoV-2 viral load and disease severity. Hildebrand *et al.* showed that pre-infection with *Mtb* 4 to 8 weeks prior to viral infection reduced the viral load in K18-hACE2 mice, which are particularly susceptible to SARS-CoV-2.^180^ The protective phenotype was also observed by other research teams.^181^^,^^182^ The authors also showed a significant reduction in bodyweight loss and viral load, not only in K18-hACE2 mice, but also in wild-type mice infected with a murine-adapted strain of SARS-CoV-2. According to Rosas-Mejia *et al.*, the resistance of *Mtb*-infected mice to SARS-CoV-2 is associated with the selective expansion of antigen-specific T cell and B cell subsets, at the expense of neutrophils, NK cells, and naive T cells.^181^ An effect on early viral replication was also observed, which was associated with reduced viral antigen burden and consequently a diminished magnitude of the T cell response.^182^ Despite notable differences in experimental models, including mouse strains, viral variants and the timing and duration of each infection, the available evidence indicates that prior *Mtb* infection may influence subsequent antiviral responses by reshaping the pulmonary immune environment to limit early viral replication and disease severity.

Human cohort studies support these experimental findings and suggest that LTBI is associated with a distinct immune response to SARS-CoV-2 infection. In an Indian cohort, coinfected individuals exhibit, compared to those without LTBI, heightened humoral, cytokine, and acute phase responses. LTBI is associated with modulation of antibody and cytokine responses and systemic inflammation in individuals seropositive for SARS-CoV-2 infection. The increased ability of LTBI individuals to produce both binding and neutralizing antibodies against SARS-CoV-2 suggests that LTBI has a protective role against SARS-CoV-2.^183^ Beyond antibody responses, alterations in interferon signaling have also been reported in patients with TB and LTBI. Reports have suggested that active TB and LTBI are associated with type I IFN production.^184^ Importantly, the timing of type I IFN responses appears to be critical for viral control, as a retrospective study of patients with COVID-19 found that early type I IFN treatment improves disease outcome, whereas late type I IFN administration is associated with increased mortality.^185^

To mechanistically investigate how *Mtb* infection might confer protection against SARS-CoV-2, several groups have investigated the immune crosstalk between *Mtb*-exposed immune cells and virus-infected targets within *in vitro* models. Using such an approach, Williams *et al.* showed that *Mtb*-exposed PBMCs can modulate epithelial cell responses to SARS-CoV-2 infection *in vitro*. In co-culture systems, SARS-CoV-2-infected cells exposed to supernatants or signals from *Mtb*-infected PBMCs displayed enhanced transcriptional activation, including expression of pro-inflammatory cytokines such as TNF-α, IL-1β, IL-6 and IFN-γ, as well as interferon-stimulated genes such as OAS1, OAS3, MX2 and IFIH1. Importantly, IFN-γ was identified as a key mediator in this process, as neutralization of IFN-γ significantly attenuated the protective effects observed in this system.^186^ In line with these findings, IFN-γ levels are elevated in the context of LTBI, indicating an enhancement and activation of the immune system.^187^

Collectively, human cohort data and mechanistic *in vitro* studies suggest that *Mtb* infection is associated with an immune milieu characterized by enhanced antiviral responsiveness, involving both humoral immunity and interferon signaling. While *in vitro* systems cannot fully recapitulate *in vivo* coinfection dynamics, they provide valuable insight into potential mediators. For instance, these findings suggest that IFN-γ may play a pivotal role in orchestrating the immune response against a multitude of pathogens, as illustrated by SARS-CoV-2,^188^ and potentially extending to other viruses such as IAV,^189^ in which it has been shown to inhibit viral attachment.

Taken together, evidence from animal models, human cohorts, and mechanistic *in vitro* studies supports the notion that chronic *Mtb* infection can establish an antiviral state that limits SARS-CoV-2 replication and disease severity. Although the underlying mechanisms remain incompletely understood, these findings suggest that *Mtb*-induced immune reprogramming may contribute to broader antiviral protection.

#### Influence of Mtb infection on systemic pathogens

TB is known to increase HIV-1 replication, as immune cell recruitment to sites of granulomatous inflammation may facilitate rapid virus propagation.^190^ Macrophages, the main target of *Mtb*, are permissive to HIV-1,^191^ and it has been shown that the anti-inflammatory macrophages present in TB microenvironments form tunneling nanotubes that allow cell-to-cell viral transfer.^192^ Moreover, HIV and *Mtb* both stimulate TNF-α production, which has been shown to enhance HIV-1 replication in macrophages.^193^ These synergistic effects are not observed when monocyte-derived macrophages are preincubated with inactivated *Mtb*, as this results in a reduction of HIV-1 replication *in vitro.*^194^

Beyond viral infections, *Mtb* has also been shown to influence systemic parasitic diseases. Similarly, Page *et al.* showed, in C57BL/6 mice, that the increased Th1 immune response caused by *Mtb* infection reduced the parasitemia of *Plasmodium yoelii*, responsible for malaria.^195^ Mice coinfected with *Mtb* and *Plasmodium berghei* exhibit exacerbated chronic TB but are less susceptible to parasitic infection.^196^ Consistent with this, recent human data support a similar trend: higher pro-inflammatory cytokines in LTBI were found to confer immunological protection against severe malaria.^177^ Collectively, these studies illustrate that *Mtb* infection can profoundly reshape systemic immune environments beyond the lung, leading to divergent outcomes during coinfection depending on the pathogen and immunological context. These findings highlight that the impact of *Mtb* infection extends far beyond the pulmonary compartment and can either enhance susceptibility or confer protection against secondary pathogens, depending on the balance of immune responses elicited during coinfection.

**Figure 3:**
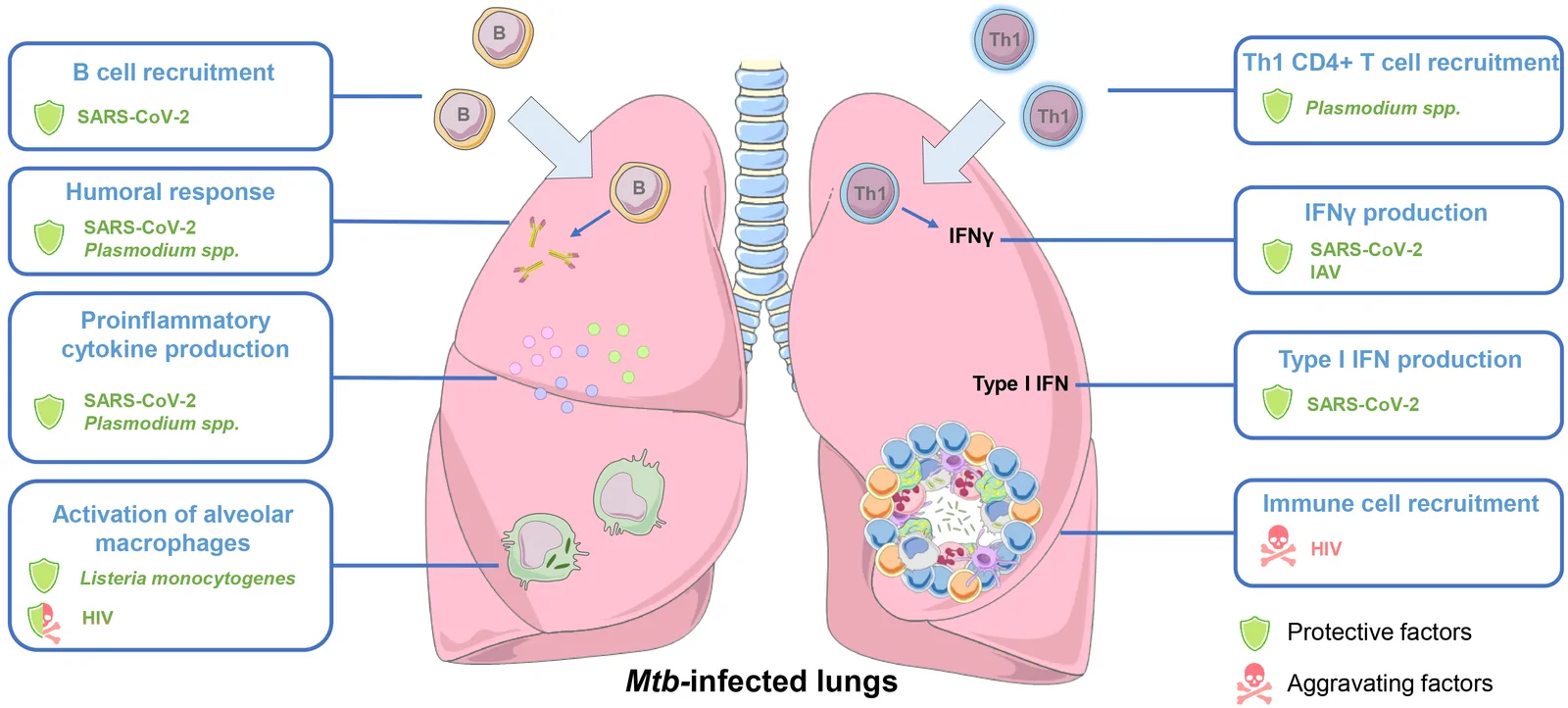
Possible modulatory effects of TB on secondary infections progression Illustration of hypothetical mechanisms by which a primary *Mtb* infection may influence the course of subsequent infections. The figure outlines how TB-induced alterations in immune cell recruitment, activation status, or cytokine production could affect the host’s response to secondary pathogens. IAV: influenza A virus; HIV: human immunodeficiency virus. The figure was partly created using Servier Medical Art templates, provided by Servier, which are licensed under a Creative Commons Attribution 4.0 Unported License (http://smart.servier.com). Icons from BioRender. Dagan, Y. (2026) (https://BioRender.com/y4mcdyo)*.*

These examples illustrate a broader pattern: the delicate immune balance required to control and maintain LTBI is often disrupted by secondary infections, which can trigger reactivation or exacerbate disease. Conversely, growing *in vitro* and *in vivo* evidence suggests that TB itself, particularly its chronic form, could provide a degree of protection against other pathogens. Yet, the underlying mechanisms behind this protective effect remain poorly understood. All the hypotheses explored in the reviewed articles are summarized in Figure 3. When combined with clinical and epidemiological observations, these findings raise important questions about the broader impact of TB on susceptibility to other infections at a global scale. Yet, such patterns remain difficult to capture due to limited data and the challenges of monitoring coinfection dynamics across diverse populations over time.

## Conclusion

Despite the availability of curative treatments and its relatively low incidence in developed countries, TB is considered a major global health threat, and many scientists have been focusing their research efforts on the development of novel, more effective therapeutic strategies. TB has been extensively shown to negatively impact comorbidities, and coinfections are no exception at first glance.

The literature reviewed here indicates that TB coinfection is frequently associated with exacerbation of secondary infections or increased host susceptibility. Through both epidemiological and experimental studies, TB coinfection leads to worsened clinical outcomes, including increased severity of viral or bacterial infections, prolonged disease progression, and higher mortality rates. Moreover, the complex immune balance that maintains *Mtb* under control can be disturbed by secondary infections, leading to reactivation and worsening of TB. Therefore, improved epidemiological surveillance of coinfections, together with integrated screening strategies for secondary pathogens in patients with TB and accurate discrimination between latent and active TB disease, could significantly improve patient outcomes. Such approaches would facilitate earlier diagnosis, better risk stratification, and more appropriate clinical management, particularly in regions where TB remains endemic and the burden of concurrent infections is high. These observations emphasize that coinfections represent a major, yet often underestimated, challenge for TB control and patient management.

Building on these insights, the therapeutic management of TB could also benefit from considering coinfections. Secondary pathogens may influence both susceptibility to TB and the progression of disease, and their presence could help explain why some individuals develop active TB following prior exposure or latent infection. Integrating this knowledge into treatment strategies, including potentially targeting both *Mtb* and coexisting pathogens, could help restore an immune balance favorable to TB control. Furthermore, a better understanding of the immunological alterations induced by coinfections may support the development of personalized host-directed or immunomodulatory therapies tailored to the patient’s immune status and infectious history. Such approaches could enhance protective immune responses while limiting detrimental inflammation. This perspective aligns with the concept of personalized medicine, where therapy is tailored not only to the infecting pathogen but also to the patient’s overall immunological and infectious profile.

However, this predominantly aggravating picture of coinfection outcomes is shaped by an important limitation of available clinical data. Patients captured in hospital-based studies are more likely to present with severe forms of TB or of the secondary infection. Consequently, clinical datasets largely reflect the most severe disease manifestations, whereas individuals with undiagnosed *Mtb* infection and mild or transient secondary infections are unlikely to seek medical care. As a result, these milder cases remain underreported, leaving a significant gap in epidemiological data and possibly leading to an underestimation of the true incidence and spectrum of TB coinfections. In addition, coinfection outcomes are strongly influenced by geographic and epidemiological factors, including regional disparities in global health, host immune status, circulating *Mtb* lineages, and exposure to distinct pathogens. These variables further shape patient populations captured in clinical studies and complicate direct comparisons across settings.

Experimental studies offer a more nuanced perspective, complementing the picture drawn from clinical and epidemiological data. *In vitro* and *in vivo* models suggest that *Mtb* infection, particularly in its chronic or latent form, could have a beneficial impact on the host’s ability to control secondary infections. This protective effect could be explained by a basal level of inflammation in the lungs and immune cell activation, creating an inhospitable environment for other pathogens. Nevertheless, demonstrating comparable protective effects in humans remains challenging due to limitations in clinical resolution, heterogeneous patient populations, and the difficulty of defining infection states and temporal relationships between pathogens. More research is needed to unravel the immune mechanisms at play in chronic *Mtb* infection and clarify how this condition might contribute to broader immune resilience and thus challenge the management of coinfections. Importantly, this will require the development of improved experimental models that more faithfully recapitulate the diversity of the TB disease spectrum, the chronicity of infection, and the complexity of host-pathogen interactions occurring during coinfections. Current models often fail to capture key features of human latent or subclinical TB and may therefore overlook mechanisms relevant to protection or disease exacerbation. In particular, future studies should integrate multi-omics approaches, including single-cell transcriptomics, epigenetic profiling, and immunometabolic analyses, to define how chronic *Mtb* infection imprints long-term immune function. Dissecting these mechanisms across tissues and disease states will be essential to understand how TB-induced immune reprogramming shapes susceptibility, protection, or pathology during coinfections.

These observations naturally connect to broader concepts of trained immunity and vaccine-induced cross-protection. Considering its protective effects on multiple infectious diseases, the use of BCG as a cross-protective vaccine against a broader spectrum of diseases could be envisaged. Interestingly, many of the pathogens against which BCG vaccination offers protection are the same as those for which chronic *Mtb* infection appears to provide a protective effect.^197^ Consequently, the mechanisms proposed to explain BCG-induced protection also serve as valuable hypotheses for understanding the protective immune profile associated with chronic *Mtb* infection.

Finally, these findings raise important questions in the context of global TB elimination strategies. The WHO aims to eradicate TB by 2050 and encourages researchers around the world to develop new therapeutic strategies, especially against multidrug-resistant *Mtb* strains.^198^ A major pillar of this plan is the treatment of LTBI, given its vast reservoir and its role as a silent driver of the TB pandemic through the lifelong risk of reactivation. Indeed, since the mechanisms underlying reactivation are still not fully understood or controllable, maintaining this reservoir is considered a major public health risk. This is further compounded by the fact that coinfections may act as facilitators of *Mtb* reactivation and progression toward subclinical or clinical disease.

As this review also highlights that chronic *Mtb* infection may confer partial immune protection against unrelated acute respiratory infections, it raises the question of whether large-scale elimination of LTBI could have unintended consequences on host immunity to other pathogens. Therefore, eliminating the risk of TB reactivation, which is particularly relevant in vulnerable populations such as the elderly or those with genetic conditions or chronic comorbidities, might have to be weighed against the potential benefits of LTBI-induced cross-protection against other respiratory illnesses. More detailed exploration and understanding of these coinfection models, together with properly designed assessment and mathematical modeling of the associated risks, are thus needed to help predict the impact of a TB-free world on the incidence of other respiratory infections.

In this context, the development of therapeutic vaccines that prevent the progression from LTBI to active disease might represent a promising compromise. Such strategies could simultaneously reduce the risk of reactivation and preserve any possible beneficial immunomodulatory effects of LTBI. Nonetheless, any future approach must be guided by precise surveillance and a better understanding of both reactivation mechanisms and the immunological interplay between LTBI and secondary infections.

## Literature search strategy

The literature included in this review was identified through searches of PubMed and Google Scholar using combinations of keywords related to tuberculosis and coinfection, including “tuberculosis,” “*Mycobacterium tuberculosis*,” “latent tuberculosis infection,” “coinfection,” “superinfection,” “secondary infection,” “viral infection,” “bacterial infection,” “fungal infection,” “parasitic infection,” “protection,” and “susceptibility.” Studies were selected based on their relevance to interactions between *Mtb* infection and secondary pathogens, as well as their contribution to understanding the underlying immunological mechanisms. We sought to include clinical, epidemiological, and experimental studies and to provide representative examples across different classes of pathogens whenever possible. Given the broad scope of the topic and the diversity of pathogens involved, the review was not intended to be exhaustive.

## Acknowledgments

Financial support for this work was provided by the 10.13039/501100001665Agence Nationale de la Recherche (N° ANR-22-CE18-0021-01) (20-PAMR-0005), and the 10.13039/501100010095Région Hauts-de-France (DOS0190627/00 – Start-AIRR BPI Program). The authors thank the European Fund for Regional Economic Development (HDF 003447), the Région Hauts-de-France, French State, Lille European Metropolis (MEL) for their financial support within the CPER RESISTOMICS program (2021-2027). This work is supported by the ANRS-MIE N° ANRS0521 (Agence Nationale de Recherche sur le Sida et les hépatites virales-Maladies Infectieuse Emergentes). Financial support for this work was also provided by the FHU Respire (CIBLe Project) and the Fondation Université de Lille. The funders had no role in study design, data collection and analysis, decision to publish, or preparation of the manuscript. ChatGPT (OpenAI, GPT-5 mini, 2026 version) and DeepL Translate (version 25.50, DeepL SE, 2026) were used to assist with language rephrasing and translation. All outputs were carefully reviewed and validated by the authors to ensure accuracy and originality. The figures were partly created using Servier Medical Art templates, provided by Servier, which are licensed under a Creative Commons Attribution 4.0 Unported License (http://smart.servier.com). The figures were partly created using BioRender. Dagan, Y. (2026) (https://BioRender.com/y4mcdyo). We are deeply grateful to Alain Baulard and Philip Supply for their careful reading of our manuscript and for their helpful advice, which greatly improved the clarity and quality of this review.

## Declaration of interests

The authors declare no competing interest.

## Supplemental information

Table S1. Major immune cells involved in TB control

Table S2. Pathogens discussed in this review and associated diseases

Table S3. Summary of epidemiological studies reporting coinfections between TB and secondary pathogens

Table S4. Summary of experimental animal studies and patients’ samples analyses exploring the interaction between TB and other pathogens
