# Supplementary material for: Unraveling the multifaceted impact of tuberculosis on coinfections
Source: iScience. 2026 Aug 12;29(8):117133. doi: 10.1016/j.isci.2026.117133 (PMC13498980; doi:10.1016/j.isci.2026.117133)
Supplement: Supplementary file 1 [file mmc1.pdf]

iScience, Volume *29*

Supplemental information

Unraveling the multifaceted impact of tuberculosis on coinfections

Joan Fine, François Trottein, Arnaud Machelart, and Valentin Sencio

Supporting Information

Supplementary Table 1

Unraveling the Multifaceted Impact of Tuberculosis on Coinfections

Joan Fine[1], François Trottein[1], Arnaud Machelart*[1], Valentin Sencio*[1]

[1]Univ. Lille, CNRS, INSERM, CHU Lille, Institut Pasteur de Lille, U1019, UMR 9017 CIIL – Center for Infection and Immunity of Lille, Lille, France

Corresponding authors: Arnaud Machelart (arnaud.machelart@inserm.fr); Valentin Sencio (valentin.sencio@cnrs.fr)

Supplementary table 1: Major immune cells involved in TB control.

Terminology	Role in TB	Key activating signals	Major mediator produced	References
Alveolar macrophages	Primary host cell for *Mtb*; initial containment or niche for persistence; orchestrate granuloma formation	IFN-γ, TNF, TLR	TNF, IL-1β, IL-6, IL-12, CXCL9, CXCL10, ROS	(Kinsella et al., 2021; Queval et al., 2017)
Monocytes	Recruited to infected lung; differentiate into macrophages or dendritic cells; contribute to inflammation and pathology	CCL2, IFN-γ	TNF, IL-1β, IL-6	(Kipnis et al., 2003; Sampath et al., 2018)
Dendritic cells	Antigen uptake and presentation; priming of *Mtb*-specific T cells in lymph nodes; early containment of the bacteria	IFN-γ, TLR signaling	IL-12, IL-6, TNF	(Mihret, 2012; Schreiber & Sandor, 2010)
Neutrophils	Early containment of the bacteria; drivers of tissue damage and disease severity	IL-8, IL-17	ROS, proteases, IL-1β	(Eum et al., 2010; Hilda et al., 2020; Hult et al., 2021)
Natural killer cells	Early IFN-γ source; cytotoxicity toward infected cells; early containment of the bacteria	IL-12, IL-15, IL-18	IFN-γ, TNF	(Allen et al., 2015; Esin & Batoni, 2014)
B cells	Antibody production; antigen presentation; immune regulation	IL-21	IL-21, CD40-CD40L	(Stewart et al., 2023)
$CD4^+$ Th1 cells	Central protective cell type; activate macrophage bactericidal functions	IL-12, IL-18	IFN-γ, TNF	(Redford et al., 2011; Sakai et al., 2014; Scanga et al., 2000)
$CD4^+$ Th17 cells	Promote neutrophil recruitment and mucosal immunity; context-dependent effects	IL-1β, IL-6, IL-23	IL-17A/F, IL-22	(Cardona & Cardona, 2019; Torrado & Cooper, 2010)
$CD4^+$ Treg cells	Limit excessive inflammation; may impair bacterial control	TGF-β, IL-2	IL-10, TGF-β	(Cardona & Cardona, 2019)
$CD8^+$ T cells	Cytotoxic killing of infected cells; contribute to bacterial control	IL-12, type I IFNs	IFN-γ, TNF, perforin, granzyme B	(Lin & Flynn, 2015)
γδ T cells	Early cytokine production; bridge innate and adaptive immunity	IL-1β, IL-23	IFN-γ, IL-17	(La Manna et al., 2020)

This table is not intended to be exhaustive and focuses exclusively on immune cell types explicitly discussed in this review. Additional cellular subsets, functional states, and context-dependent roles described elsewhere in the tuberculosis literature are therefore not included.
