# Supplementary material for: Unraveling the multifaceted impact of tuberculosis on coinfections
Source: iScience. 2026 Aug 12;29(8):117133. doi: 10.1016/j.isci.2026.117133 (PMC13498980; doi:10.1016/j.isci.2026.117133)
Supplement: Supplementary file 2 [file mmc2.pdf]

Supporting Information

Supplementary Table 2

Unraveling the Multifaceted Impact of Tuberculosis on Coinfections

Joan Fine[1], François Trottein[1], Arnaud Machelart*[1], Valentin Sencio*[1]

[1]Univ. Lille, CNRS, INSERM, CHU Lille, Institut Pasteur de Lille, U1019, UMR 9017 CIIL – Center for Infection and Immunity of Lille, Lille, France

Corresponding authors: Arnaud Machelart (arnaud.machelart@inserm.fr); Valentin Sencio (valentin.sencio@cnrs.fr)

Supplementary table 2: Pathogens discussed in this review and associated diseases

Pathogen	Type of pathogen	Associated disease (host)	Route of transmission	Primary site of infection	References
Cytomegalovirus	Virus (DNA, Herpesviridae)	Usually latent, severe systemic disease in immunocompromised hosts (humans)	Bodily secretions, organ transplantation	Systemic (hematopoietic cells, multiple organs)	(Dioverti & Razonable, 2016)
Hepatitis virus	Virus (RNA, Flaviviridae)	Chronic hepatitis (liver inflammation) (humans)	Bodily fluids (mostly blood)	Liver (hepatocytes)	(Castaneda et al., 2021)
Human immunodeficiency virus	Virus (RNA, Retroviridae)	Acquired immunodeficiency syndrome (weakened immune system) (humans)	Blood-borne and sexual transmission	Immune cells ($CD4^+$ T cells, macrophages)	(Bekker et al., 2023)
Influenza virus	Virus (RNA, Orthomyxoviridae)	Influenza (fever, cough, pneumonia) (humans and animal models)	Respiratory droplets/aerosols	Respiratory tract (epithelial cells)	(Moghadami, 2017)
Lymphocytic choriomeningitis virus	Virus (RNA, Arenaviridae)	Fever, meningitis, chronic immune activation (mice)	Contact with infected rodents	Systemic (spleen, liver, central nervous system)	(Vilibic-Cavlek et al., 2021)
Measles virus	Virus (RNA, Paramyxoviridae)	Measles (fever, cough, rash, transient immunosuppression) (humans)	Respiratory droplets/aerosols	Respiratory tract (cells expressing CD150)	(Moss & Griffin, 2024)
SARS-CoV-2	Virus (RNA, Coronaviridae)	COVID-19 (cough, pneumonia, ARDS, systemic inflammation) (humans)	Respiratory droplets/aerosols	Respiratory tract (epithelial cells)	(Uraki et al., 2026)
Brucella abortus	Bacterium (intracellular)	Brucellosis (fever, fatigue, hepatosplenomegaly) (humans and animals)	Ingestion, contact with animals	Reticuloendothelial system (macrophages, monocytes, dendritic cells)	(Dorneles et al., 2015)
Chlamydia trachomatis	Bacterium (obligate intracellular)	STIs, trachoma, urethritis, cervicitis, conjunctivitis (humans)	Sexual transmission	Urogenital tract, conjunctiva	(Murray & McKay, 2021)
Haemophilus influenzae	Bacterium	Respiratory infections, otitis, bronchitis, pneumonia (humans)	Respiratory droplets	Upper and lower respiratory tract	(MacAlasdair et al., 2025)
Helicobacter hepaticus	Bacterium	Hepatitis, colitis, intestinal inflammation, cofactor of hepatic cancer (mice)	Fecal-oral transmission	Liver, intestine	(Falsafi & Mahboubi, 2013)

Helicobacter pylori	Bacterium	Gastritis and peptic ulcer disease (humans)	Oral-oral and fecal-oral transmission	Stomach	(Malfertheiner et al., 2023)
Klebsiella pneumoniae	Bacterium	Pneumonia and systemic infections (humans)	Respiratory transmission and contact	Respiratory tract	(Abbas et al., 2024)
Listeria monocytogenes	Bacterium (intracellular)	Listeriosis (fever, gastroenteritis, meningitis) (humans and animal models)	Food-borne	Systemic (liver, spleen, central nervous system)	(Radoshevich & Cossart, 2018)
Mycobacterium leprae	Bacterium	Leprosy (skin lesions, neuropathy) (humans)	Prolonged close contact	Skin, peripheral nerves	(Grijsen et al., 2024)
Streptococcus pneumoniae	Bacterium	Pneumonia, meningitis, bacteremia (humans)	Respiratory droplets	Respiratory tract	(Narciso et al., 2025)
Schistosoma mansoni	Parasite (helminth)	Intestinal schistosomiasis (diarrhea, hepatosplenic disease) (humans)	Skin penetration (freshwater)	Mesenteric vasculature, intestine	(Anderson & Enabulele, 2021)
Plasmodium berghei	Parasite (protozoan)	Experimental malaria (fever, anemia) (mice)	Vector-borne (mosquito)	Liver, red blood cells	(Gozalo et al., 2024)
Plasmodium falciparum	Parasite (protozoan)	Severe malaria (fever, anemia, cerebral malaria) (humans)	Vector-borne (mosquito)	Liver, red blood cells	(Heide et al., 2019)
Plasmodium yoelii	Parasite (protozoan)	Experimental malaria (fever, anemia)	Vector-borne (mosquito)	Liver, red blood cells	(Gozalo et al., 2024)
Aspergillus fumigatus	Fungus (filamentous)	Pulmonary aspergillosis (cough, pulmonary infiltrates, invasive disease) (humans)	Inhalation of spores	Respiratory tract	(van de Veerdonk et al., 2025)
Candida albicans	Fungus	Candidiasis (oral/genital thrush, systemic infection) (humans)	Endogenous overgrowth	Mucosae, bloodstream	(Talapko et al., 2021)

This table includes pathogens that are either directly discussed in the main text or specifically addressed in dedicated studies cited in this review (see Supplementary table 1 and 2). It is not intended to be exhaustive, as additional pathogens may be mentioned or investigated within the referenced articles. The sole purpose of this table is to clarify the diseases and organ systems associated with each pathogen.
