# Supplementary material for: Unraveling the multifaceted impact of tuberculosis on coinfections
Source: iScience. 2026 Aug 12;29(8):117133. doi: 10.1016/j.isci.2026.117133 (PMC13498980; doi:10.1016/j.isci.2026.117133)
Supplement: Supplementary file 3 [file mmc3.pdf]

Supporting Information

Supplementary Table 3

Unraveling the Multifaceted Impact of Tuberculosis on Coinfections

Joan Fine[1], François Trottein[1], Arnaud Machelart*[1], Valentin Sencio*[1]

[1]Univ. Lille, CNRS, INSERM, CHU Lille, Institut Pasteur de Lille, U1019, UMR 9017 CIIL – Center for Infection and Immunity of Lille, Lille, France

Corresponding authors: Arnaud Machelart (arnaud.machelart@inserm.fr); Valentin Sencio (valentin.sencio@cnrs.fr)

Supplementary table 3: Summary of epidemiological studies reporting coinfections between TB and secondary pathogens

Secondary pathogens	Authors	Type	Year of publication	Country	Year of study	Secondary pathogen	Objective/hypothesis	Number studied	TB diagnosis	Findings	Protection/ exacerbation	DOI/PMID
**Cytomegalovirus**	Kua et al. [95]	Meta-analysis	2021	International	2003-2021	Cytomegalovirus	To investigate whether prior CMV infection is associated with higher risk of developing TB disease	15 articles/38 618 patients	Varied by study	Pooled findings show that individuals with CMV infection have higher risk of TB disease.	Exacerbation (TB)	DOI: 10.1093/infdis/jiac179
**Cytomegalovirus**	Martinez et al. [97]	Prospective birth cohort study	2021	South Africa	2012-2021	Epstein-Barr virus, Cytomegalovirus	To determine whether CMV acquisition in early infancy increases the risk of developing TB disease during childhood	1,143 children enrolled	TST, X-ray, GeneXpert, culture and respiratory samples	CMV infection before 6 weeks of age was associated with a significantly higher risk of TB disease later in childhood.	Exacerbation (TB)	DOI: 10.1016/S2214-109X(21)00407-1
**Cytomegalovirus**	Stockdale et al. [96]	Case-control study	2019	Uganda	1989-2018	Epstein-Barr virus, Cytomegalovirus, herpes simplex virus	To assess whether elevated anti-CMV IgG levels are associated with increased future risk of active TB disease	25 TB cases and 256 non-TB controls	Previous clinical or microbiological diagnosis	Higher CMV IgG antibody levels predicted increased odds of developing active TB disease.	Exacerbation (TB)	DOI: 10.1093/infdis/jiz581
**Cytomegalovirus**	van der Heijden et al. [94]	National survey analysis	2021	USA	1999-2016	Cytomegalovirus	To assess whether CMV seropositivity is associated with higher prevalence of LTBI in the US	NHANES dataset (sample size not specified)	QuantiFERON-TB Gold In-Tube (QFT-GIT) positivity	CMV infection is linked to higher prevalence of latent TB, suggesting potential exacerbating effect. CMV and LTBI both linked to higher C-reactive protein.	Exacerbation (TB)	DOI: 10.1093/ofid/ofab539
**Hepatitis C virus**	Baliashvili et al. [99]	Population based cohort-study	2023	Georgia	2015-2020	Hepatitis C virus	To evaluate whether chronic HCV infection, treated or untreated, is associated with increased risk of active TB disease	1,828,808 adults tested for HCV, 3,163 active TB	National program records (clinical and microbiological confirmation)	Chronic HCV infection significantly increases risk of active TB, especially if untreated.	Exacerbation (TB)	DOI: 10.1093/cid/ciac786
**Hepatitis C virus**	Behzadifar et al. [98]	Meta-analysis	2019	International	2000-2018	Hepatitis C virus	To determine the prevalence of HCV infection among TB patients and highlight the importance of HCV screening in this population	8 studies/14,676 patients	Varied by study	Pooled prevalence of HCV in active TB patients was 9%, while latent/active combined was 2%. HCV co-infection is common among TB patients and increases risk of anti-TB drug hepatotoxicity, indicating need for routine HCV testing.	Exacerbation (TB)	DOI: 10.4314/ejhs.v29i1.17
**Hepatitis B virus**	Malden et al. [102]	Retrospective cross-sectional analysis	2023	USA	2008-2019	Hepatitis B virus	To describe screening patterns and identify risk factors for coinfection with LTBI and HBV in a large healthcare system	1,997 HBV patients screened for LTBI; 35,820 LTBI	LTBI defined by TST or IGRA (QuantiFERON) positivity	Among HBV patients, 23.1 % had LTBI; among LTBI patients, 1.3 % had HBV. HBV and LTBI co-infection is prevalent in high-risk groups.	Exacerbation (TB/HBV)	DOI: 10.1016/j.amjmed.2023.10.031

								patients screened for HBV				
**Hepatitis B and C viruses**	Nooredinvand et al. [101]	Prospective observational study	2015	UK	2008-2011	Hepatitis B virus, hepatitis C virus	To determine the prevalence of HBV and HCV infection in patients starting anti-TB therapy	429 patients (270 active TB; 159 latent TB)	Clinical examination prior to anti-TB treatment; methods not detailed	Active TB group had higher prevalence of chronic viral hepatitis markers (16/270 vs 2/159 for LTBI patients).	Exacerbation (HBV/HCV)	DOI: 10.3748/wjg.v21.i29.8920
**Hepatitis C virus**	Wu et al. [100]	Population-based cohort study	2015	Taiwan	1999-2007	Hepatitis C virus	To determine whether HCV infection is associated with a higher risk of developing active TB disease	5,454 HCV-infected individuals and 54,274 age- and sex-matched non-HCV controls	National health insurance records	TB incidence rate was higher in HCV group: 134.1 vs. 89.1 per 100,000 persons-years.	Exacerbation (HCV)	DOI: 10.1097/MD.0000000000001328
**Human immunodeficiency virus**	Daley et al. [81]	Outbreak cohort study	1992	USA	1990-1991	HIV-1	To compare lung granuloma architecture and TNF production between TB/HIV-1 coinfected and TB-only patients	30 exposed, 11 developed active TB + 28 staff	Culture-positive *M. tuberculosis*, tuberculin skin test conversions	Among exposures, 37 % (11/30) developed active TB; 13 % had new TST conversions. HIV infection dramatically accelerates progression from recent TB infection to active disease.	Exacerbation (TB)	DOI: 10.1056/NEJM199201233260404
**Human immunodeficiency virus**	de Noronha et al. [79]	Retrospective autopsy cohort	2008	Brazil	1995-2001	HIV-1	Comparison of lung granuloma architecture and TNF production between TB/HIV-1 coinfected and TB-only patients	5 HIV/TB coinfected and 4 TB-only autopsy cases	PCR and histology postmortem	Coinfected granulomas were poorly formed, necrotic, rich in neutrophils; they had more bacillary load and greatly reduced TNF staining.	Exacerbation (TB)	DOI: 10.1016/j.prp.2007.10.008
**Human immunodeficiency virus**	Kusejko et al. [92]	Observational cohort analysis	2020	Switzerland	1999-2020	HIV-1	To assess whether LTBI is associated with lower HIV set-point viral load and reduced incidence of opportunistic infections in people with HIV	13,943 HIV-positive participants (840 with LTBI; 770 with active TB)	TST and IGRA testing	LTBI was associated with a lower HIV viral set-point and lower odds of candida stomatitis and oral hairy leukoplakia compared to *Mtb*-uninfected HIV positive individuals.	Protection (HIV)	DOI: 10.1371/journal.pbio.3000963
**Human immunodeficiency virus**	Garfein et al. [84]	Cross-sectional field study	2010	Mexico	April-July 2007	HIV-1	To estimate LTBI prevalence and associated HIV co-infection risk factors among marginalized high-risk groups	503 participants	IGRA test	High LTBI prevalence among HIV-exposed groups, indicating elevated TB reactivation risk with HIV spread.	Exacerbation (TB)	DOI: 10.3201/eid1605.091446

**Human immunodeficiency virus**	Geldmacher et al. [80]	Cross-sectional longitudinal cohort study	2008	Tanzania	No explicitly stated	HIV-1	To assess the effect of HIV-1 infection (acute and chronic) on *Mtb*-specific CD4+ Th1 responses	114 HIV-negative and 68 HIV-positive, 5 latently infected individuals	Cellular response to RD1 antigens in the absence of clinical symptoms.	Chronic HIV+ individuals had significantly reduced *Mtb*-specific Th1 responders.	Exacerbation (TB)	DOI: 10.1086/593017
**Human immunodeficiency virus**	Sonnenberg et al. [82]	Retrospective cohort study	2005	South Africa	Between 1990s and 2000s	HIV-1	To quantify how soon after HIV infection the risk of developing pulmonary TB increases	23,874 miners including 740 TB cases analyzed	Chest-radiograph screening, contact tracing, sputum smear examination	TB incidence: 0.80/100 person-years at risk in HIV-negative vs. 2.90/100 in HIV-positive miners; TB risk doubled within first year after seroconversion.	Exacerbation (TB)	DOI: 10.1086/426827
**Human immunodeficiency virus**	Sullivan et al. [86]	Observational cohort study	2015	South Africa	No explicitly stated	HIV-1	To assess whether LTBI or active TB contributes to systemic immune activation in HIV-infected individuals	80 HIV-positive individuals with well-defined states of TB	TST, ESAT-6/CFP-10 specific IFNγ ELISpot, sputum	Active TB patients had elevated monocyte activation markers; both LTBI and active TB groups showed elevated T-cell activation, suggesting that LTBI drives immune activation and increases risk of progression to AIDS.	Exacerbation (HIV)	DOI: 10.1016/j.ebiom.2015.03.005
**Human immunodeficiency virus**	Toossi et al. [85]	Observational cohort study	2001	Uganda	1995-2000s	HIV-1	To investigate whether active TB at the pleural site enhances in situ HIV-1 replication and identify potential immune mediators	30 HIV-1/TB patients	Chest-radiograph diagnosis	Pleural biopsy showed significant HIV-1 replication at the site of active *Mtb* infection in HIV-1/TB patients.	Exacerbation (HIV)	DOI: 10.1097/00042560-200109010-00001
**Human immunodeficiency virus**	Xun et al. [87]	Prospective cohort study	2020	China	No explicitly stated	HIV-1	To determine whether TB co-infection increases size of HIV reservoir (total HIV DNA) in PBMCs after anti-retroviral therapy (ART)	13 HIV/TB coinfected; 10 HIV-only controls	*Mtb* smear/culture	TB co-infection is associated with larger HIV reservoir, potentially complicating HIV eradication efforts.	Exacerbation (HIV)	DOI: 10.1186/s12981-020-00320-0
**Influenza virus**	Abadom et al. [103]	Case-population study	2016	South Africa	2009-2012	Seasonal and pandemic: A (H1N1)/A(H3N2) and B	To identify underlying medical conditions linked to increased risk of influenza-associated hospitalization	3,646 subjects	Microscopy, *Mtb* culture/XPert *MTB*/RIF	TB is a risk factor for influenza-associated hospitalization.	Exacerbation (influenza virus)	DOI: 10.1016/j.vaccine.2016.09.011
**Influenza virus**	Archer et al. [106]	Epidemiological surveillance report	2009	South Africa	2009	HIV-1, A(H1N1)pdm09	To describe epidemiology, mortality, and risk factors, including HIV and TB, for fatal pandemic H1N1 cases	442 subjects	Microscopy, *Mtb* culture/XPert *MTB*/RIF	10% cases who died with influenza had TB, against 1% TB prevalence in general community (not evaluated statistically).	Exacerbation (influenza virus)	DOI: 10.2807/ese.14.42.19369-en

**Influenza virus**	De Paus et al. [108]	Retrospective case–control serological study	2013	Indonesia	2001-2004	Seasonal A(H3N2)/A(H1N1)	To assess whether recent influenza infections contribute to active TB development	111 TB cases, 111 controls	Clinical + CXR changes positive microscopy and culture for *Mtb*	Prevalence of influenza antibodies among TB cases was 46% vs 41% for healthy controls.	Neutral	DOI: 10.1016/j.tube.2013.02.006
	Dube et al. [113]	Observational immunological cohort study	2016	South Africa	2011-2012	Viruses (hMPV, influenza A, B and C, CMV, coronavirus), bacteria (*Moraxella catarrhalis, Streptococcus pneumoniae, Haemophilius influenzae, Staphilococcus aureus, Mycoplasma pneumoniae, Bordetella pertussis, Chlamydia pneumoniae*)	To detect and compare respiratory pathogens in the nasopharynx of children with confirmed vs suspected TB	214 subjects (34 TB positive)	*Mtb* culture/XPert *MTB*/RIF	High prevalence of polymicrobial carriage: no significant difference in respiratory pathogen detection between TB and non-TB groups. Influenza was found in 18% of TB positive children vs 4% for TB negative.	Neutral	DOI: 10.1186/s12879-016-1934-z
**Influenza virus**	Koegelenberg et al. [109]	Prospective observational ICU cohort study	2009	South Africa	2009	A(H1N1)pdm09	To describe epidemiology, management, comorbidities, and outcomes of severe H1N1 influenza in ICU patients	19 cases	Not specified	2/19 patients with influenza also had TB, and both died.	Neutral	DOI: 10.1093/qjmed/hcq022
**Influenza virus**	Noh et al. [110]	Retrospective case series	2013	South Korea	2009-2011	A(H1N1)pdm09	To describe clinical characteristics and timing of active TB diagnosis in patients with confirmed influenza A(H1N1)pdm09	12,196 subjects	TB PCR and culture	No deaths in the 7 cases of concurrent TB/influenza infection.	Neutral	DOI: 10.3201/eid1901.111613
**Influenza virus**	Ope et al. [111]	Matched case–control study	2011	Kenya	2007-2009	HIV-1, Seasonal A(H3N2)/A(H1N1)/B	To identify risk factors (including HIV and pulmonary TB) for influenza-associated hospitalization	64 cases, 190 controls	Self-report, verified by clinician diagnosis	TB correlates with influenza hospitalization on bivariate but not on multivariate analysis.	Neutral	DOI: 10.1371/journal.pone.0020111
**Influenza virus**	Puvanaligam et al. [107]	Retrospective clinical case series	2011	India	2009-2010	A(H1N1)pdm09	To describe clinical characteristics, comorbidities (including TB), and outcomes of H1N1 cases	442 H1N1-positive cases	Recorded patient history	Pulmonary TB was a significant risk factor for complications in H1N1 infection.	Exacerbation (influenza virus)	PMID: 21751659

**Influenza virus**	Roth et al. [112]	Hospital-based surveillance	2013	Thailand	2003-2011	Seasonal and pandemic A(H1N1)	To determine the frequency and severity of concurrent influenza and TB in hospitalized patients with respiratory illness	7,180 subjects	≥1 sputum Acid Fast Bacilli or culture positive	Death in 0/23 cases with co-infections vs 30/646 in TB only patients.	Neutral	DOI: 10.1111/j.1750-2659.2012.00413.x
**Influenza virus**	Umbreen et al. [104]	Prospective cohort study	2023	Pakistan	No explicitly stated	A(H1N1)pdm09	To estimate the burden of influenza A(H1N1)pdm09 infection among TB-positive patients compared to the general population	152 with TB, 152 controls	Clinical diagnosis	TB cohort had higher incidence of influenza (25.5/1000) vs 15.2/1000 in controls.	Exacerbation (influenza virus)	DOI: 10.1186/s12879-023-08441-3
**Influenza virus**	Walaza et al. [105]	Prospective hospital-based active surveillance cohort	2015	South Africa	2010-2011	A(H1N1)pdm09/A (H3N2)/B	To determine whether influenza coinfection increases mortality among hospitalized pulmonary TB patients	2,959 subjects	Microscopy, *Mtb* culture/XPert *MTB*/RIF	Increased risk of death in cases with co-infection vs TB only.	Exacerbation (TB)	DOI: 10.1186/s12879-015-0746-x
**Measles virus**	Lee et al. [132]	Population-based retrospective cohort study	2008	South Korea	2000-2004	Measles (paramyxovirus)	To evaluate whether measles infection increases subsequent incidence of TB	53,974 measles cases: 47 subsequent TB cases	National TB registry reports	Measles did not elevate TB risk; possibly associated with a modest reduction in post-measles TB incidence.	Neutral/Protection (TB)	DOI: 10.1086/528856
**SARS-CoV-2**	Inoue & Kashima [135]	Ecological (population-level) correlation analysis	2021	International	1900 (TB incidence), 1950 (TB mortality), 2020 (COVID-19 data)	SARS-CoV-2	To test whether past TB epidemics are associated with lower COVID-19 incidence and mortality	90 countries (1990 data); 28 countries (1950 data)	Historical national TB incidence/mortality metrics	Countries with high past TB burden had lower COVID-19 incidence and mortality, suggesting possible long-term trained immunity.	Protection (SARS-CoV-2)	DOI: 10.1371/journal.pone.0253169
**SARS-CoV-2**	Takahashi [136]	Hypothesis/econometric analysis using instrumental variables	2020	International	Historical LTBI prevalence via WHO estimates, 2020 (COVID-19 mortality)	SARS-CoV-2	To test if national LTBI prevalence is inversely associated with COVID-19 mortality	90 countries	WHO country-level data estimations	LTBI prevalence was significantly associated with reduced COVID-19 mortality in adjusted instrumental variable analysis.	Protection (SARS-CoV-2)	DOI: 10.1016/j.mehy.2020.110214
	Huaman et al. [129]	Population-based retrospective cohort study	2015	USA	2000-2011	HIV-1, *Chlamydia trachomatis, Salmonella spp., Shigella spp., Yersinia spp., Listeria monocytogenes*	To determine if TB patients have increased risk of subsequent intracellular bacterial infections	3,214 TB cases	Culture, smear, clinical	No increased risk of intracellular bacterial infections, and notably fewer chlamydia infections soon after TB diagnosis; some Salmonella excess was HIV-related rather than TB-driven.	Protection (*Chlamydia spp.*)	DOI: 10.1017/S0950268814002131
Haemophilus influenzae	Martynova et al. [114]	Cross-sectional hospital/primary-care observational study	2011	Russia	No explicitly stated	*Haemophilius influenzae*	To determine the frequency of *H. influenzae* carriage in children with TB and characterize isolates	85 children	Not explicitly stated	Frequent *H. influenzae* carriage among TB-affected children; microbiological characterization suggesting opportunistic colonization.	Exacerbation (*H. influenzae*)	DOI: 10.1155/2011/420284

	Mhimbira et al. [130]	Cross-sectional household-controlled cohort study	2018	Tanzania	2013-2015	Respiratory viruses (human rhinovirus A/B/C, influenza A, respiratory syncytial virus A/, adenovirus, parainfluenza virus 2/3/4, coronavirus 226E/OC34/NL63, enterovirus, bocavirus 1/2/3/4, metapneumovirus), bacteria (*Haemophilus influenzae, Streptococcus pneumoniae, Legionella pneumophila, Bordetella parapertussis, Mycoplasma pneumoniae, Bordetella pertussis, Chlamydophila pneumoniae*)	To compare prevalence of respiratory viruses/bacteria in TB patients versus household contacts and assess clinical impact	794 participants (489 TB patients, 305 household controls)	Sputum culture/Xpert or clinical diagnosis	Co-detection of bacterial and viral pathogens in TB patients linked to more severe pulmonary disease. However, respiratory bacteria were less likely be detected in TB patients than in controls.	Exacerbation (respiratory bacteria)	DOI: 10.1016/j.cmi.2018.03.019
	Shimazaki et al. [138]	Prospective hospital-based observational cohort study	2018	Philippines	2011-2013	*Streptococcus pneumoniae, Haemophilus influenzae, Moraxella catarrhalis, Mycoplasma pneumoniae, Chlamydia pneumoniae, Legionella pneumophila*	To determine prevalence of bacterial coinfection in hospitalized pulmonary TB patients and assess its effect on early mortality	466 hospitalized patients including 228 TB patients	PCR on sputum	29% (135/466) had bacterial pathogens in sputum (21% *H. influenzae*, 7.9% *S. pneumoniae*). Bacterial coinfection increases early mortality risk in pulmonary TB patients.	Exacerbation (TB)	DOI: 10.5588/ijtld.17.0389

	Attia et al. [115]	Retrospective cross-sectional cohort study	2019	Cambodia	2012-2013	*Klebsiella spp., Pseudomonas spp., Escherichia coli, Enterobacter spp., Staphylococcus aureus, Haemophilus influenzae, Acinetobacter baumanii, Burkholderia pseudomallei, Burkholderia maltophila*	To determine prevalence and etiologies of bacterial coinfection among respiratory infection patients tested for TB and other bacteria	137 patients	Sputum smear, culture (MGIT/LJ) and/or Xpert *MTB*/RIF	40 (29%) had TB; 60 (44%) had bacterial infection; 13 (9% of all, 33% of TB patients) had both. Bacterial co-infection frequent in TB patients (1 in 3) and associated with radiologic evidence of cavitation, suggesting worsened pulmonary disease.	Exacerbation (respiratory bacteria)	DOI: 10.1186/s12890-019-0828-4
	Bir et al. [137]	Cross-sectional observational study	2024	India	2023	*Pseudomonas aeruginosa, Acinetobacter baumannii, Klebsiella pneumoniae, Stenotrophomonas maltophila*	To determine the prevalence and profile of bacterial coinfections in microbiologically confirmed pulmonary TB patients	174 pulmonary TB patients	Xpert *MTB*/RIF Ultra	Bacterial respiratory coinfections are common (~11%) and involve resistant pathogens not targeted by TB therapy, underscoring the need for co-pathogen screening.	Exacerbation (TB/respiratory bacteria)	DOI: 10.7759/cureus.66482
Klebsiella spp.	Liu et al. [140]	Retrospective hospital-based cohort study	2023	China	2023	*Klebsiella pneumoniae*	To describe clinical and microbiological characteristics of *K. pneumoniae* coinfections in TB patients	76 TB patients with *K. pneumoniae* coinfection	Culture, smear, GeneXpert	Multi-drug resistant *K. pneumoniae* coinfection leads to increased respiratory compromise, inflammation, and need for medical support alongside TB.	Exacerbation (TB/*K. pneumoniae*)	DOI: 10.2147/IDR.S421587
	Pujiaty et al. [139]	Retrospective cross-sectional hospital-based cohort study	2024	Indonesia	2020-2022	*Klebsiella pneumoniae, Enterobacter cloacae, Acinetobacter baumannii, Pseudomonas aeruginosa, Escherichia coli, Aeromonas hydrophila, Stenotrophomonas maltophilia, Pandoraea spp., Sphingomonas paucimobilis, Enterococcus*	To characterize bacterial/fungal coinfection profiles and drug sensitivity patterns in pulmonary TB	125 pulmonary TB patients	GeneXpert *MTB*/RIF and sputum culture	33.6% had bacterial growth, 8.8% fungal, 30.4% mixed; *K. pneumoniae* and *C. albicans* most common.	Neutral	DOI: 10.21010/Ajidv19i1.3

						casseliflavus, Granulicatella adiacens, Staphylococcus aureus, Staphylococcus haemolyticus, Staphylococcus spp., Streptococcus constellatus, Streptococcus mitis, Streptococcus sanguinis, Streptococcus parasanguinis, Rothia mucilaginosa						
Mycobacterium leprae	Cardenas et al. [128]	Cross-sectional pilot study	2016	USA	2014	*Mycobacterium leprae*	To evaluate prevalence and potential cross-immunity between LTBI and asymptomatic leprosy infection	25 volunteers	Enzyme-linked immunospot assay (T.SPOT)	The presence of leprosy antibodies was associated with lower likelihood of LTBI, supporting potential cross-protective immunity.	Protection (*M. leprae*)	PMID: 27255065
**Nontuberculous mycobacteria**	Lin et al. [148]	Population-based retrospective cohort	2020	Taiwan	2020	Nontuberculous mycobacteria (not explicitely specified)	To estimate incidence rates of NTM disease and NTM–TB coinfection, and assess demographic and socioeconomic factors	3,148 TB patients, 450 NTM patients, among which 87 NTM-TB coinfected patients	ICD-9-CM, laboratory codes, and treatment records	NTM–TB co-infection, though uncommon (2.8%), complicates TB diagnosis and treatment, especially in older adults.	Neutral	DOI: 10.1097/MD.0000000000023775
**Nontuberculous mycobacteria**	López-Varela et al. [147]	Prospective cohort study	2017	Mozambique	2011-2012	*M. intracellulare, M. scrofulaceum, M. fortuitum, M. malmoense, M. interjectum, M. gordonae, M. chelonae, M. abscessus*	To determine prevalence and clinical impact of NTM isolation among children evaluated for TB	775 children with presumptive TB	Standard pediatric presumptive criteria	NTM isolated in 26.3% of children, and their presence did not worsen clinical outcomes of TB disease.	Neutral	DOI: 10.1371/journal.pone.0169757

Streptococcus pneumoniae	Agwu et al. [141]	Cross-sectional observational study	2006	Nigeria	Not explicitly stated	*Streptococcus pneumoniae*	To assess prevalence of *S. pneumoniae* infection among patients attending TB clinics	330 sputum samples tested	Acid Fast Bacilli microscopy (Ziehl-Neelsen stain)	*S. pneumoniae* detected in 21/330 (6.4%); *Mtb* in 81/330 (24.5%).	Neutral	/
Streptococcus pneumoniae	Isaac et al. [133]	Cross-sectional observational study	2023	Nigeria	2019	*Streptococcus pneumoniae*	To determine prevalence of S. pneumoniae and its antimicrobial resistance among patients with pulmonary infections, including TB patients	273 sputum samples	GeneXpert *MTB*/RIF assay	The occurrence rate of *S. pneumoniae* and *Mtb* co-infections is low among suspected pulmonary infection cases with an occurrence rate of 1.10%.	Protection (*S. pneumoniae*)	DOI: 10.216 08/mid. 2022.1 38402. 1314
Streptococcus pneumoniae	Samson et al. [90]	Cross-sectional observational study	2019	Nigeria	2018	HIV-1, *Streptococcus pneumoniae*	To determine the prevalence and antimicrobial sensitivity patterns of *S. pneumoniae* and *Mtb* coinfection in HIV-positive adults on retroviral treatment	260 participant s	Acid Fast Bacilli microscopy (Ziehl-Neelsen stain)	Although co-infection was relatively uncommon (2.7%), the presence of *S. pneumoniae* alongside antimicrobial resistance stresses the importance of integrated bacterial and TB screening among HIV patients.	Exacerbation (TB)	DOI: 10.239 37/246 9-567X/1 510048
Streptococcus pneumoniae	Schleicher & Feldman [89]	Retrospective case series	2003	South Africa	2003	HIV-1, *Streptococcus pneumoniae*	To describe occurrence and clinical features of coinfection with *S. pneumoniae* and *Mtb* infections in HIV-positive adults	9 cases of dual infections	Microbiological diagnosis	Coinfection noted in HIV-positive individuals may delay TB diagnosis and complicate treatment.	Exacerbation (TB)	PMID: 146778 97
**Helminth**	Brown et al. [127]	Prospective cohort analysis	2006	Uganda	Not explicitly stated	HIIV-1, *Mansonella perstans, Schistosoma mansoni*	To evaluate whether helminth infections increase the risk of progression to active TB among HIV-positive adults	462 patients	TST	No significant association for helminths overall; however, *S. mansoni* infection was associated with higher risk of active TB.	Neutral/Exac erbation (TB)	PMID: 166876 87
**Helminth**	Elias et al. [125]	Case-control study	2006	Tanzania	1999-2002	*Ascaris lumbricoides, Hookworm, Strongyloides stercoralis, Schistosoma mansoni, Trichuris trichiura, Enterobius vermicularis*	To determine if intestinal helminth infection is associated with active pulmonary TB	230 TB patients and 510 healthy household contact	Sputum smear-positive	Helminth prevalence reaches 71% in TB cases vs 36% in contacts.	Neutral	

**Helminth**	Neto et al. [126]	Retrospective cohort via clinical record review	2009	Brazil	2000-2006	*Strongyloides stercoralis, Entamoeba histolytica, Giardia lamblia*	To measure prevalence of intestinal parasitism in pulmonary TB	327 TB patients	Smear/culture/TST	19.6% of TB patients had enteroparasites; eosinophilia correlated with parasitism, but no significant impact on TST positivity or survival	Neutral	DOI : 10.1590/s1413-86702009000600008
**Helminth**	Tristão et al. [124]	Retrospective case-control study	2002	Brazil	1997-1999	*Ascaris lumbricoides, Strongyloides stercoralis, Trichuris trichiura, Necator americanus, Ancylostoma duodenale, Schistosoma mansoni*	To assess whether intestinal nematode infection is more prevalent among TB patients and could modulate immune response	57 pulmonary TB patients and 86 matched non-TB controls	Not explicitly stated	Nematode infection in 57.8% of TB patients vs 20.9% of controls.	Exacerbation (nematode)	DOI: 10.1590/S0037-86822002000500020
**Helminth**	Zenebe et al. [123]	Meta-analysis	2023	International	2007-2022	*Ascaris lumbricoides,* Hookworm, *Strongyloides stercoralis, Schistosoma mansoni, Trichuris trichiura, Hymenolepis nana, Taenis spp., Enterobius vermicularis*	To quantify prevalence of intestinal helminth co-infection in TB patients and identify associated risk factors	22 studies	Varied by study	*S. mansoni* and *S. stercoralis* had the highest pooled prevalence among TB cases. Only *S. stercoralis* had significant association with TB cases compared to TB negatives.	Neutral/Exacerbation (helminths)	DOI: 10.1186/s12879-023-08716-9
Leishmania donovani	el-Safi et al. [146]	Community-based cross-sectional study	2004	Sudan	2002	*Leishmania donovani*	To determine individual-level overlap between *L. donovani* and *Mtb* infections	382 individuals	TST	66% leishmanin skin test-positive, 26% TST-positive, and 20% positive for both. No significant association between the two infections at individual level after adjusting for age.	Neutral	DOI: 10.1111/j.1365-3156.2004.01337.x

	Range et al. [120]	Cross-sectional observational study	2007	Tanzania	2005-2006	HIV-1, *Schistosoma haematobium, Schistosoma mansoni,* Hookworm, *Plasmodium spp., Ascaris lumbricoides, Trichuris trichiura, Strongyloides stercoralis*	To determine the prevalence of parasitic co-infections among confirmed or suspected pulmonary TB patients and examine associations with HIV status	655 patients including 532 TB patients	Sputum microscopy/culture confirmation	34% of TB patients infected with *S. mansoni*, 18% with hookworms, 3.2% had malaria parasitemia.	Neutral	DOI: 10.1179/136485907X176373
Plasmodium spp.	Teixeira et al. [122]	Ecological spatial analysis	2019	Brazil	2012-2015	*Plasmodium vivax, Plasmodium falciparum*	To assess if local malaria incidence is spatially associated with TB incidence, controlling for socio-economic/environmental factors	All municipalities from the Amazon state	Reported notified TB cases per municipality	Spatial clustering suggests malaria burden is positively linked with higher TB incidence; individual-level coinfections are probable.	Exacerbation (TB)	DOI: 10.1111/tmi.13282
Plasmodium spp.	Valadas et al. [121]	Retrospective clinical study	2013	Angola	2007	HIV-1, *Plasmodium falciparum*	To characterize coinfection rates of TB with malaria and HIV, and assess associated hospital outcomes	1,906 TB patients	Clinical and radiological findings, Mantoux test, sputum microscopy	Malaria and HIV co-infections were common and associated with elevated in-hospital mortality among TB patients.	Exacerbation (HIV, *Plasmodium spp.*)	DOI: 10.3855/jidc.2703
Aspegillus spp.	Dellière et al. [145]	Retrospective hospital-based cohort study	2020	France	2011-2017	*Aspergillus fumigatus, Aspergillus nigri, Aspergillus terrei, Aspergillus flavi, Aspergillus versicolor, Aspergillus chevalieri*	To assess prevalence, clinical characteristics, and significance of co-isolation of *Mycobacterium* and *Aspergillus spp.* in respiratory specimens	50 coinfected patients	Culture methods	*Mycobacterium* and *Aspergillus* are frequently co-isolated, but active coinfection is uncommon; most *Aspergillus* findings are colonizing.	Neutral	DOI: 10.3389/fmicb.2019.02980
Aspegillus spp.	Hosseini et al. [144]	Meta-analysis	2020	International	2001-2019	*Aspergillus fumigatus*	To estimate prevalence of pulmonary *Aspergillus* coinfection among patients with pulmonary TB	17 studies	Varied by study	15.4% of TB patients had culture or serology evidence of *Aspergillus spp.*, but studies did not consistently link *Aspergillus* presence to worse clinical outcomes.	Neutral	DOI: 10.1016/j.micpath.2020.104018

	Yadu et al. [143]	Observational cohort study	2015	India	2006-2007	*Candida albicans, Candida parapsilosis, Aspergillus fumigatus, Aspergillus niger, Fusarium solani, Rhizopus oryzae, Rhodotorula mucilaginosa, Geotrichum candidum, Myridontium keratinophylum*	To estimate prevalence of fungal coinfection among patients with pulmonary TB	100 patients with pulmonary TB	Acid Fast Bacilli microscopy (Ziehl-Neelsen stain)	49% of TB patients were positive for fungal infection, with mostly *C. albicans* (34.69%), *C. parapsilosis* (26.53%) and *A. fumigatus* (12.24%).	Neutral	/
Candida spp.	Amala et al. [142]	Cross-sectional observational study	2020	Nigeria	2018-2019	*Candida albicans, Candida parapsilosis, Candida tropicalis, Candida krusei*	To determine prevalence of *Candida spp.* coinfection and antifungal susceptibility patterns among TB patients	400 sputum from TB patients	Not specified	Over one-third (34.4%) of TB patients showed *Candida* coculture, suggesting opportunistic colonization/infection.	Neutral	DOI: 10.4236/jtr.2020.82006
	Astekar et al. [118]	Cross-sectional observational study	2016	India	Not explicitly stated	*Candida albicans, Candida tropicalis, Candida krusei, Candida parapsilosis, Candida glabrata, Candida lusitaniae, Candida nivariensis, Aspergillus spp.*	To assess prevalence and species of fungal infections in pulmonary TB patients	60 patients with pulmonary TB	Ziehl-Neelsen staining	55% of TB patients had *Candida spp.* isolated, and 5% had *Aspergillus spp.*	Neutral	DOI: 10.4103/0973-029X.185913
Candida spp.	Kali et al. [116]	Cross-sectional observational study	2013	India	2010-2011	*Candida albicans, Candida tropicalis, Candida krusei, Candida parapsilosis, Candida glabrata*	To determine prevalence and species distribution of *Candida* coinfection in pulmonary TB patients	75 patients with pulmonary TB	Acid Fast Bacilli microscopy (Ziehl-Neelsen stain)	40% of TB patients had *Candida spp.*, including *C. albicans* (50%), *C. tropicalis* (20%) and *C. glabrata* (20%).	Neutral	DOI: 10.4066/AMJ.2013.1709
	Wu et al. [117]	Retrospective cohort study	2025	China	2022-2024	*Candida albicans, Candida tropicalis, Candida krusei, Candida parapsilosis, Candida glabrata, Aspergillus spp.*	To identify prevalence, characteristics, and risk factors for fungal co-infection among TB patients	218 TB patients, including 87 with fungal infections	Clinical symptoms and chest X-ray/CT scan	39.9% had fungal coinfection, predominantly with *C. albicans* (42.7%), *C. tropicalis* (20.6%) and *C. glabrata* (17.6%). Coinfected cases had more cough, fever, anemia, rales, and significantly lower serum protein and hemoglobin levels	Exacerbation (fungi)	DOI: 10.62347/CBWV2685

Pneumocystis carinii	Aderaye et al. [88]	Cross-sectional study	2003	Ethiopia	2001-2002	HIV-1, *Pneumocystis jirovecii*	To determine prevalence of *P. carinii* in HIV-positive TB suspects	119 HIV-positive TB-negative patients;96 HIV-positive TB-positive	Sputum culture or smear	High prevalence of *P. carinii* among HIV/TB coinfected suspects.	Exacerbation (*P. carinii*)	DOI: 10.1097/00002030-200302140-00018
