# Supplementary material for: Unraveling the multifaceted impact of tuberculosis on coinfections
Source: iScience. 2026 Aug 12;29(8):117133. doi: 10.1016/j.isci.2026.117133 (PMC13498980; doi:10.1016/j.isci.2026.117133)
Supplement: Supplementary file 4 [file mmc4.pdf]

Table S4: Summary of experimental animal studies and patients' samples analyses exploring the interaction between TB and other pathogens.

Secondary pathogens	Authors	Type	Year of publication	Direction of interaction	Objective/ hypothesis	Experiment settings	Secondary pathogen strain	Period/ observation period	Findings	Protection/ Exacerbation	DOI/PMID
**Cytomegalovirus**	Müller et al. [161]	Human samples	2019	CMV → TB	To determine if CMV infection and CMV-induced immune activation increase the risk of subsequent TB disease in infants.	Case-control study including 49 infants who developed TB and 129 matched controls. PBMC were assessed for CMV-stimulated IFNγ response via ELISpot, CD8+ T cell activation, NK cell gene signature and whole blood transcriptional profiles.	Cytomegalovirus, Epstein-Barr virus	CMV and EBV screened at 4-6 months of age, and tracking for first TB diagnosis up to 2 years.	CMV-specific IFNγ response is associated with increased CD8+ T cell activation and CMV-positive infants have increased risk of developing TB disease. They also have lower expression of NK cells and a lower frequency of CD3- CD4- CD8- lymphocytes.	Exacerbation (TB)	DOI: 10.1172/jci.insight.130090
**Human immunodeficiency virus**	Bucşan et al. [158]	Non-human primate model	2019	SIV → TB	To determine whether SIV-induced CD4⁺ T cell depletion is the main driver of reactivation of LTBI in coinfected macaques.	39 rhesus macaques infected with aerosolized *Mtb* to establish LTBI. After 9 weeks, infection with SIV and monitoring for TB reactivation via clinical signs, imaging, bacterial burden and immune profiling. A group of macaques was treated with CD4R1 antibodies to deplete CD4+ T cells.	SIVmac239, SIVΔGY	LTBI established for 9 weeks. Macaques followed up to 18 weeks post-SIV infection or antibody treatment until necropsy.	Pathogenic SIVmac239 induced reactivation, but non-depleting SIVΔGY or CD4R1-induced CD4+ T cell depletion alone failed to cause reactivation.	Exacerbation (TB)	DOI: 10.1172/JCI125810
**Human immunodeficiency virus**	Corleis et al. [157]	Human samples /Non-human primate model	2019	HIV-1/SIV → TB	To assess whether infection by HIV-1 or SIV leads to loss of lung interstitial CD4⁺ T cells and consequently increases dissemination of pulmonary *Mtb*.	*Ex vivo* experiments using lymphocytes collected from human lungs, tonsils and blood, and *in vivo* experiments using humanized mice and rhesus macaques. Use of non-human primate coinfection model to assess dissemination of *Mtb* following early stage SIV infection.	CCR5-tropic HIV-1 (for human samples/mice experiments), SIV (for macaque experiments)	Mice experiments: 4 to 7 weeks post-coinfection; macaque experiments: LTBI established for 9 weeks before SIV coinfection for 11-13 weeks.	Rapid and severe loss of lung interstitial CD4+ T cells in all models, associated with increased dissemination of pulmonary *Mtb* in macaque model.	Exacerbation (TB)	DOI: 10.1016/j.celrep.2019.01.021
**Human immunodeficiency virus**	Diedrich et al. [156]	Non-human primate model	2010	SIV → TB	To investigate whether SIV infection reactivates LTBI in macaques and determine whether reactivation correlates with viral load or early T cell depletion.	Cynomolgus macaques with LTBI infected with SIV, with a monitoring of peripheral and airway T cell counts, virus levels and TB reactivation indicators.	SIVmac251	6 to 8 months to establish LTBI, follow up to 11 months post-SIV infection. Early reactivation defined as <17 weeks.	All macaques with LTBI reactivated after SIV infection, regardless of viral load. Early reactivation was associated with more pronounced peripheral and airway CD4+ T cell loss.	Exacerbation (TB)	DOI: 10.1371/journal.pone.0009611

**Human immunodeficiency virus**	Goletti et al. [194]	Cellular model	2004	TB → HIV-1	To evaluate whether exposure to *Mtb* or its purified protein derivative (PPD) modulates HIV-1 replication in human macrophages.	Human monocyte-derived macrophages were infected with HIV-1, then treated with live *Mtb* or PPD. HIV-1 replication was assessed by measuring viral p24 antigen production.	CCR5-tropic HIV-1	HIV-1 added after 48 h of incubation with *Mtb*	Exposure to live *Mtb* or PPD significantly inhibited HIV-1 replication in macrophages.	Protection (HIV)	DOI: 10.1086/381554
**Human immunodeficiency virus**	Souriant et al. [192]	Cellular model/ Non-human primate model	2019	TB → HIV-1	To investigate how *Mtb*–induced microenvironments enhance HIV-1 infection in macrophages via IL-10/STAT3–dependent tunneling nanotube formation.	Human monocytes were differentiated into macrophages, treated with conditioned media from *Mtb*-infected macrophages or pleural effusions from TB patients, then infected with HIV-1 . Monitored HIV p24 levels and infection index.	CCR5-tropic HIV-1	Conditioned media added 3 days before HIV-1 infection, then follow up to 13 days.	TB-induced IL-10/STAT3 signaling axis activation results in higher levels of HIV-1 p24 and infection index. Infected macrophages formed tunneling nanotubes, facilitating direct cell-to-cell HIV-1 transfer. Blocking IL-10, STAT3, or TNT formation significantly reduced HIV-1 spread and replication.	Exacerbation (HIV)	DOI: 10.1016/j.celrep.2019.02.091
**Human immunodeficiency virus**	Tepekule et al. [93]	Human samples	2024	TB → HIV-1	To determine how asymptomatic *Mtb* infection in people living with HIV alters PBMC transcriptomic profiles and whether these immune perturbations correlate inversely with HIV-1 viremia, possibly explaining improved antiretroviral control during *Mtb* infection.	Peripheral blood mononuclear cells from the Swiss HIV Cohort (*Mtb*+/- and or HIV viremic/suppressed) were transcriptomically profiled. Findings were validated using an independent African cohort. Functional pathways (e.g., IL-6, TNF, KRAS) were analyzed.	HIV-1	Single time-point sampling.	*Mtb* infection induces distinct PBMC transcriptional shifts independent of viral suppression. Inflammatory pathways (IL-6, TNF, KRAS) are modulated. A strong inverse correlation was observed between *Mtb*-associated gene signature and HIV-1 viral load, both at individual gene and composite signature level.	Protection (HIV)	DOI: 10.1016/j.isci.2024.111228
**Influenza virus**	Mendy et al. [159]	Human samples	2019	Influenza → TB	To determine whether influenza co-infection at TB diagnosis alters *Mtb* burden and immune profiles in patients.	Analyzed sputum samples from 282 active TB patients for influenza A/B RNA and quantitation of *Mtb* via 16s rRNA. Cytokines were assessed using multiplex assay. PBMCs analyzed by flow cytometry.	Influenza A and B	At TB diagnosis (single time point).	4.3% (12/282) of active TB patients were coinfected with influenza A/B, with higher sputum bacterial loads. Lower IL-17A levels in sputum and higher MCP-1 in PPD-stimulated blood.	Exacerbation (TB)	DOI: 10.3389/fimmu.2018.03093
**Influenza virus**	Redford et al. [160]	Mice model	2014	Influenza → TB	To determine the effect of prior IAV infection on susceptibility and control of TB, through a type I interferon receptor-dependent pathway.	Mice were intranasally infected with IAV before aerosol challenge with *Mtb* (model 1). Alternatively, *Mtb*-infected mice were superinfected with IAV post-TB infection (model 2). Outcomes measured included lung bacterial load, survival curves,	Influenza A X31 or PR8	Model 1: *Mtb* aerosolization 28 days post-IAV infection. Follow up to 120 days post-TB infection; model 2: IAV superinfection 1 and 14 days after *Mtb* infection. Follow up to	Model 1 shows significant increase in inflammation, decreased survival and higher CFU count in lung compared to the control group. In model 2, coinfected mice had significantly increased mycobacterial load	Exacerbation (TB)	DOI: 10.1093/infdis/jit424

						and assessment in type I IFN receptor knockout mice.		27 days post-IAV infection.	compared to the control group. The detrimental effect was dependent on the type I IFN receptor as knock-out mice did not show impaired TB control.		
**Influenza virus**	Ring et al. [164]	Mice model	2019	Influenza → TB	To determine whether influenza A virus coinfection exacerbates tuberculosis by impairing control of *Mtb* via IL-10–mediated immunomodulatory mechanisms.	Mice were aerosol-infected with *Mtb*, then intranasally infected with Influenza A post-*Mtb* infection. Bacterial loads in lung and spleen, survival, CD4⁺ T cell responses, macrophage polarization, and lung cytokine were monitored. Therapeutic blockade of IL-10 receptor signaling was assessed using anti–IL-10R antibody.	Influenza A virus (A/HH/05/09 H1N1)	IAV infection at day 12 post-*Mtb* infection. Endpoint assessments on days 1, 3, 9 post-IAV; survival monitored for 4 weeks post-*Mtb*.	IAV coinfection led to significantly increased *Mtb* lung and spleen bacterial burdens and reduced survival compared to *Mtb*-alone mice. Coinfected mice showed impaired induction of *Mtb*-specific CD4⁺ T cells, and IL-10 production was rapidly induced by IAV. Blocking IL-10 receptor signaling restored *Mtb* control to levels similar to *Mtb*-only infected mice.	Exacerbation (TB)	DOI: 10.1172/jci.insight.126533
**Lymphocytic choriomeningitis virus**	Kang et al. [172]	Mice model	2022	LCMV → TB	To elucidate how viral coinfection prior to pulmonary infection of *Mtb*-specific Th1 cells impairs TB control by inducing type I IFN signaling.	Mice were infected via aerosol with *Mtb* then coinfected with LCMV. Lung *Mtb* burden, histopathology and CD4+ Th1 cell influx were assessed. To test rescue of immune response, type I IFN receptor were blocked.	Lymphocytic choriomeningitis virus	LCMV infection at day 14 post-*Mtb* infection. Follow up to 17 days post-LCMV infection. Anti-IFNAR1 treatment at day -1, 1 and 3 post-LCMV infection.	Viral coinfection induced sustained type I IFN signaling, suppressing lung CXCL9/CXCL10 expression in myeloid cells. The impaired pulmonary recruitment of *Mtb*-specific TH1 CD4+ T cells results in higher bacterial loads and lung immunopathology. Blocking type I IFN receptor signaling restored chemokine production, Th1 cell influx, and improved TB control.	Exacerbation (TB)	DOI: 10.1038/s41467-022-30914-3
**Lymphocytic choriomeningitis virus**	Xu et al. [174]	Mice model	2022	LCMV → TB	To investigate how a pre-existing chronic viral infection alters the pulmonary immune environment to worsen subsequent *Mtb* infection.	Mice were first chronically infected with LCMV to mimic persistent viral infection, then challenged with aerosolized *Mtb*. Bacterial loads, survival and pulmonary dissemination are monitored. Mechanistic studies focused on TNFα-mediated early arrest of *Mtb*, dendritic cell migration, CD4⁺ T cell priming, Th1 /Th17 skewing, and neutrophil accumulation.	Lymphocytic choriomeningitis virus	*Mtb* infection 21 days post-LCMV infection. Follow up to 301 days.	LCMV increased *Mtb* burden, extrapulmonary spread and mortality. Elevated TNFα in lungs initially arrested *Mtb* growth but impaired dendritic cell migration to lymph nodes, delaying CD4⁺ T cell priming. CD4 T cell differentiation shifted from Th1 to Th17, increasing neutrophil infiltration and reducing long-term survival. Restoring timely CD4⁺ T cell priming improved TB control.	Exacerbation (TB)	DOI: 10.1016/j.immuni.2021.01.003

**SARS-CoV-2**	Baker et al. [182]	Mice model	2022	SARS-CoV-2 → TB, TB → SARS-CoV-2	To determine whether SARS-CoV-2 coinfection affects *Mtb* infection outcome and whether pre-existing *Mtb* infection influences early SARS-CoV-2 replication.	K18-hACE2 mice were infected intranasally with SARS-CoV-2 one month before aerosol *Mtb* challenge, or infected with *Mtb* before SARS-CoV-2 exposure. C57Bl/6 mice were coinfected with the Beta variant of SARS-CoV-2 post-*Mtb*. Outcomes included weight, lung pathology, bacterial loads in lungs/spleen and early viral titers in lungs post-SARS-CoV-2.	SARS-CoV-2 USA-WA1/2020 and Beta variant (B.1.351)	*SARS-CoV-2* infection 1-month post-*Mtb* challenge. *Mtb* burden and pathology assessed at 4 and 20 weeks post-*Mtb* challenge.	Pre-existing *Mtb* infection limited early SARS-CoV-2 replication in lungs. SARS-CoV-2 infection did not exacerbate *Mtb* disease, with no differences in bacterial burden, weight loss, or lung pathology compared to *Mtb*-only controls.	Protection (SARS-CoV-2)	DOI: 10.3389/fimmu.2023.1240419
**SARS-CoV-2**	Hildebrand et al. [180]	Mice model	2022	SARS-CoV-2 → TB	To determine whether SARS-CoV-2 superinfection alters immunity to existing *Mtb* infection, affecting bacterial loads, inflammation, and dissemination.	Mice infected with *Mtb* were superinfected with SARS-CoV-2. Measured SARS-CoV-2 viral loads, survival, TB bacterial loads in lung/spleen/liver, histopathology, and cytokine profiles.	SARS-CoV-2 USA-WA1/2020	Superinfection occurred at 4- or 8-weeks post-*Mtb*; outcomes assessed 4–7 days post-SARS-CoV-2.	Prior *Mtb* infection resulted in lower SARS-CoV-2 lung and spleen viral loads. However, SARS-CoV-2 superinfection increased dissemination of *Mtb* (notably splenic loads) and reduced lung histiocytic inflammation.	Protection (SARS-CoV-2)/Exacerbation (TB)	DOI: 10.1128/spectrum.03075-22
**SARS-CoV-2**	Rajamanickam et al. [183]	Human samples	2021	TB → SARS-CoV-2	To evaluate whether LTBI modulates humoral, cytokine, chemokine, acute phase, and growth factor responses in individuals seropositive for SARS-CoV-2.	Comparison of 61 SARS-CoV-2 seropositive individuals with LTBI vs. 72 without LTBI. Measured SARS-CoV-2 specific IgM, IgG, IgA, neutralizing activity; plasma cytokines, chemokines, acute phase proteins, growth factors.	SARS-CoV-2	Single time-point sampling during SARS-CoV-2 seroconversion	LTBI patients exhibited higher plasma levels of anti-SARS-CoV-2 IgM, IgG, IgA and neutralizing antibodies. Plasma levels of IFN-γ, IL-2, TNF-α, IL-1α/β, IL-6, IL-12, IL-15, IL-17, IL-3, GM-CSF, IL-10, IL-25, IL-33, CCL3, CXCL10, CRP, α-2 macroglobulin, VEGF, and TGF-α were also elevated in LTBI individuals.	Neutral	DOI: 10.1016/j.jinf.2021.07.029
**SARS-CoV-2**	Rosas Mejia et al. [181]	Mice model	2022	TB → SARS-CoV-2	To determine whether chronic *Mtb* infection confers resistance to secondary SARS-CoV-2 infection and whether SARS-CoV-2 affects *Mtb* burden.	Mice aerosol-infected with *Mtb* (90 CFU); 30 days later challenged intranasally with SARS-CoV-2 (25,000 PFU) in transgenic and mouse-adapted models. Monitored weight change, viral/bacterial lung loads at days 4, 7, and 14 post-challenge, and scRNA-seq on lung immune cells.	SARS-CoV-2 USA-WA1/2020 and mouse-adapted MACoV2 strain MA10	SARS-CoV-2 infection 30 days post-*Mtb* infection. Follow up to 2 weeks. Assessments made at day 4, 7 and 14 post SARS-CoV-2 infection.	*Mtb*-infected mice did not lose weight after SARS-CoV-2 challenge, unlike virus-only controls. Lower viral loads in lungs of coinfected mice versus virus-only. SARS-CoV-2 infection did not affect *Mtb* burden in lungs, spleen, or liver. Lung scRNA-seq revealed expansion of T and B cell subsets, suggesting an *Mtb*-conditioned lung environment to resist SARS-CoV-2 infection.	Protection (SARS-CoV-2)	DOI: 10.1371/journal.ppat.1010093

**SARS-CoV-2**	Williams et al. [186]	Cellular model/ Mice model	2024	TB → SARS-CoV-2	To understand whether chronic *Mtb* infection primes the innate immune system via cytokine-mediated upregulation of antiviral molecules, thereby reducing SARS-CoV-2 severity.	*In vivo*: mice infected with *Mtb*, then challenged with SARS-CoV-2; monitored survival, weight, lung viral titers, and pathology. *In vitro*: Supernatants from *Mtb*-infected human PBMCs applied to SARS-CoV-2–infected epithelial cells; measured viral replication and induction of interferon-stimulated genes.	SARS-CoV-2 USA-WA1/2020	*In vivo*: outcome measures including survival and viral load post-viral challenge up to 14 days. *In vitro*: epithelial cells treated with PBMC supernatant for 24 h then infected; viral replication assessed over 48 h.	*Mtb*-infected mice exhibited reduced SARS-CoV-2 severity (improved survival, less weight loss, and lower viral titers compared to virus-only controls). *Mtb*-conditioned PBMC supernatants conferred antiviral state on epithelial cells, reducing viral replication and inducing interferon-stimulated genes expression. IFN-γ identified as a key mediator.	Protection (SARS-CoV-2)	DOI: 10.3389/fimmu.2024.1424374
Brucella abortus*, *Listeria monocytogenes	Mackaness [57]	Mice model	1964	TB → *L. monocytogenes, B. abortus*	To explore whether infection by pathogens like *Mtb* induces a state of non-specific cellular resistance in mice, mediated by activated macrophages, and to understand mechanisms involving antigen-specific antibody and delayed-type hypersensitivity.	Mice were infected with live *L. monocytogenes*, *B. abortus*, or *Mtb*. Following antigenic sensitization, passive transfer experiments evaluated macrophage activation: presence of antigen and antibody on macrophage surfaces was tested, alongside assays of macrophage-mediated resistance across different infections.	*Listeria monocytogenes, Brucella abortus*	Short-term immune activation following primary infection; exact duration not specified, focusing on acute phase post-infection.	Infection with *Mtb* (and other pathogens) induced a non-specific state of resistance, relying on activated macrophages. This resistance required both immunological reactivity and pathogen-specific antigens present in tissues.	Protection (*L. monocytogenes, B. abortus*)	DOI: 10.1084/jem.120.1.105
Helicobacter spp.	Majlessi et al. [162]	Mice model	2017	*H. hepaticus* → TB	To determine whether chronic colonization by *H. hepaticus* modulates gut microbiota and systemic immunity, and negatively affects control of a subsequent *Mtb* infection.	B10.D2 mice were gavaged with *H. hepaticus* to establish chronic gut colonization, then aerosol-challenged with *Mtb*. Measure of lung and bacterial loads, lung histopathology, immune cell infiltration, cytokine levels and gut microbiota composition.	*Helicobacter hepaticus* strain 3B1	2 months of *H. hepitacus* colonization before TB challenge, then outcomes assessed 8 weeks post-TB challenge.	*H. hepaticus* colonization led to major alterations in gut microbiota and induced subclinical inflammation, increased lung bacterial burden and exacerbated lung pathology.	Exacerbation (TB)	DOI: 10.1038/mi.2016.140
Helicobacter spp.	Perry et al. [163]	Human samples /Non-human primate model	2010	*H. pylori* → TB	To test whether chronic *H. pylori* infection enhances host immune responses against *Mtb* and is associated with reduced progression from latent to active TB.	339 healthy individuals assessed for LTBI and *H. pylori* seropositivity, then measured *Mtb*-specific IFN-γ and Th1 cytokine responses. Confirmed with Pakistan and Gambia cohorts. Cynomolgus macaques model: naturally H. pylori+ or - before *Mtb challenge.*	*Helicobacter pylori*	Human immunoassays at one time point; TB progression tracked over 2 years; macaque TB outcome at 6–8 months post-*Mtb* challenge	*H. pylori* positive individuals with latent TB had higher *Mtb*-specific IFN-γ and Th1 cytokines. In macaques, *H. pylori*+ animals had a lower risk of active TB.	Protection (TB)	DOI: 10.1371/journal.pone.0008804

Listeria monocytogenes	Jespersen [59]	Mice model	1976	TB → *L. monocytogenes*	To test whether vaccination with *Mtb* induces protective immune cross-resistance against *L. monocytogenes* in mice.	Mice were vaccinated with *Mtb* strains, then challenged intravenously with *L. monocytogenes*. Outcomes included survival rates and bacterial clearance over several weeks post-challenge.	*Listeria monocytogenes*	*L. monocytogenes* challenge 3 weeks post-immunization, then follow up to 10 weeks post-challenge.	Vaccination with *Mtb* strains conferred significant resistance to *L. monocytogenes*, demonstrating prolonged survival and enhanced bacterial clearance, whereas unvaccinated controls had high mortality.	Protection (*L. monocytogenes*)	DOI: 10.1111/j.1699-0463.1976.tb01956.x
Listeria monocytogenes	Nemeth et al. [178]	Mice model	2020	TB → TB re-challenge, *L. monocytogenes*	To test whether a contained, persistent, non-pathogenic *Mtb* infection confers rapid and durable protection against subsequent *Mtb* exposure and *L. monocytogenes* by reprogramming innate immunity.	Mice were infected with low-dose aerosolized *Mtb* to establish a contained infection, followed by a re-challenge via aerosol with virulent *Mtb*. Comparison of the bacterial burden, innate immune responses (alveolar macrophage activation and T cell recruitment) in mice with a contained *Mtb* infection vs naive mice.	*Mycobacterium tuberculosis, Listeria monocytogenes*	28 days for establishment of contained *Mtb,* follow up to 100 days post-*Mtb* aerosol challenge. CFU for *L. monocytogenes* measured after 48 h.	Contained *Mtb* infection led to rapid and sustained reduction in bacterial burden upon re-exposure.	Protection (TB re-challenge, *L. monocytogenes*)	DOI: 10.1371/journal.ppat.1008655
**Helminth**	Babu et al. [150]	Human samples	2009	Helminths → TB	To investigate whether filarial coinfection modulates Th1 and Th17 responses specific to *Mtb* in individuals with LTBI.	PBMCs from purified protein derivate-positive individuals coinfected with filarial parasites or not were stimulated with purified protein derivate or *Mtb* antigens. Cytokine secretion and expression of regulatory molecules were measured.	Filarial parasites (unspecified species)	Single time-point sampling; stimulation of PBMCs for 24 h.	Filarial coinfection significantly diminished *Mtb*-specific Th1 and Th17 cytokine responses, associated with an increase of CTLA-4 and PD-1 expression.	Exacerbation (TB)	DOI: 10.1086/599797
**Helminth**	Chatterjee et al. [170]	Human samples	2015	Helminths → TB	To assess whether filarial coinfection impairs *Mtb*-specific memory T cell compartments.	PBMCs from 25 filarial-infected and 14 filarial-uninfected LTBI subjects were stimulated with filarial antigen and *Mtb*-specific CFP-10. Multiparameter flow cytometry was used to measure IL-4 production in $CD4^+$ memory subsets.	Filarial parasites (unspecified species) antigens (BmA)	Single time-point sampling; overnight stimulation of PBMCs.	Filarial coinfection significantly increased frequencies of IL-4-producing $CD4^+$ memory T cells in response to both filarial antigen and *Mtb*-specific CFP-10. This shift toward Th2-type profile may impair Th1-mediated *Mtb* control.	Exacerbation (TB)	DOI: 10.4049/jimmunol.1402718
**Helminth**	George et al. [165]	Human samples	2013	Hookworms → TB	To determine whether coincident hookworm infection suppresses protective Th1 and Th17 cellular responses in individuals with LTBI.	Whole blood or PBMC from individuals with latent TB, with or without hookworm infection, were stimulated with *Mtb* antigens. Functional $CD4^+$ T cell subsets and regulatory T cell frequencies were assessed by flow cytometry.	*Ascaris, Strongyloides, Trichuris, Enterobius, Taenia and Hymenolepsis spp.*	6 h of *Mtb* antigens stimulation.	Hookworm coinfection was associated with significant reduction in Th1 and Th17 $CD4^+$ T cell subsets, suggesting that hookworms dampen protective cellular immunity against LTBI.	Exacerbation (TB)	DOI: 10.4049/jimmunol.1203311

**Helminth**	George et al. [168]	Human samples	2014	*W. bancrofti, S. stercoralis* → TB	To determine if helminth coinfection suppresses $CD4^+/CD8^+$ Th1 and Th17 responses and related cytokines in active TB via IL-10 mediated mechanisms.	Whole blood from TB patients, with or without helminth infection, were stimulated ex vivo with mycobacterial antigens or anti-CD3. Mono- and multifunctional Th1/Th17 $CD4^+$ and $CD8^+$ T cells and plasma cytokines were assessed.	*Wuchereria bancrofti, Strongyloides stercoralis*	6 h of *Mtb* antigens stimulation.	Coinfected patients exhibited significant reduction in antigen-specific mono- and multifunctional Th1 and Th17 $CD4^+/CD8^+$ T cells.	Exacerbation (TB)	DOI: 10.1371/journal.ppat.1004375
**Helminth**	George et al. [166]	Human samples	2015	*S. stercoralis* → TB	To assess how helminth co-infection alters systemic cytokine responses in cases of active and latent tuberculosis.	Samples from 88 individuals with active TB, including 42 infected with *S. stercoralis*, were assessed for plasma levels of type 1, type 17, type 2, regulatory, and other pro-inflammatory cytokines.	*Strongyloides stercoralis*	Single time-point sampling.	Type 1/17 cytokines were significantly reduced in coinfected individuals, while regulatory and type 2 cytokines were increased.	Exacerbation /Protection (TB)	DOI: 10.1016/j.tube.2015.09.009
**Helminth**	O'Shea et al. [151]	Human samples	2018	*S. stercoralis* → TB	To investigate whether asymptomatic hookworm coinfection affects LTBI prevalence and whether it influences functional control of *Mtb in vitro*	Screened 645 adult Nepalese migrants for LTBI and intestinal helminths, measured *in vitro* mycobacterial growth inhibition in whole blood and PBMC before and after anti-helminthic treatment.	*Strongyloides stercoralis*	Follow-up 3 to 6 months post-helminth treatment. Whole blood samples inoculated with *Mtb* for 96 h.	Hookworm infection was associated with significantly lower odds of LTBI. Pre-treatment blood samples showed enhanced mycobacterial growth inhibition *in vitro*, an effect which diminished post-helminth treatment.	Exacerbation /Protection (TB)	DOI: 10.3389/fimmu.2018.02893
**Helminth**	Potian et al. [153]	Mice model	2011	*N. brasiliensis* → TB	To determine if preexisting helminth infection impairs early innate immunity against airborne *Mtb* via Th2-type responses engaging the IL-4 receptor pathway.	Mice infected with N. brasiliensis then aerosol-challenged with *Mtb.* Evaluation of pulmonary bacterial burden, survival, macrophage polarization, and used IL-4Rα knockout mice as controls.	*Nippostrongylus brasiliensis*	*Mtb* infection 5 days after *N. brasiliensis* larvae infection	Preexisting helminth infection caused transient impairment in resistance to *Mtb*, with higher lung bacterial loads. $IL\text{-}4R\alpha^{-}/^{-}$ mice resisted this impairment.	Exacerbation (TB)	DOI: 10.1084/jem.20091473
**Helminth**	Resende Co et al. [167]	Human samples	2007	*A. lumbricoides /T. trichiura/S. stercoralis* → TB	To evaluate the effect of asymptomatic intestinal helminth coinfection on anti-*Mtb* immunity and clinical outcomes during TB treatment.	Measurement of baseline cell subset counts and whole-blood IFNγ and IL-10 after *Mtb* culture filtrate stimulation on samples from TB patients with or without helminth coinfections.	*Ascaris lumbricoides, Trichuris trichiura, Strongyloides stercoralis*	Single time-point sampling.	Helminth infection led to dampened anti-TB immunity and worse clinical outcomes, with lower frequency of immune cells, lower IFNγ and higher IL-10 compared to TB-only patients.	Exacerbation (TB)	DOI: 10.1111/j.1365-2249.2006.03247.x

**Helminth**	Talaat et al. [149]	Cellular model	2006	*B. malayi* → TB	To examine whether pre-exposure to filarial microfilariae modifies dendritic cell and macrophage responses to subsequent *Mtb* infection.	Human monocytes were differentiated into dendritic cells and macrophages, pre-exposed to live *B. malayi* microfilariae then infected with *Mtb*. The expression of surface markers and capacity to stimulate autologous CD4+ T cells were measured.	*Brugia malayi*	Cells were infected with microfilariae 2 days before *Mtb* infection.	Cells exposed to microfilariae show reduced expression of activation and costimulatory proteins, and suppress key innate immune responses required for effective anti-TB immunity.	Exacerbation (TB)	DOI: 10.1086/498912
**Helminth**	Togarsimalemath et al. [152]	Cellular model	2021	Helminth antigens → TB	To examine whether exposure to helminth antigens enhances innate immune control of M. tuberculosis in human phagocytic cells	PBMCs or isolated monocytes/macrophages were pre-exposed to protein antigens of *A. lumbricoides* or *S. mansoni*, then infected by *Mtb*. Assessed bacterial growth in monocytes and macrophages and activation markers/T-cell cytokines.	Antigens from *Ascaris lumbricoides* and *Schistosoma mansoni*	PBMC model: 20 h of antigen exposure then 5 days of *Mtb* infection; long term exposure model: monocyte exposed to conditioned medium for 8 days then 5 days of *Mtb* incubation; tissue macrophage model: monocyte-derived macrophages exposed to conditioned medium before 5 days of *Mtb* incubation	Pre-exposure to helminth antigens reduced *Mtb* growth in monocytes and macrophages. No change in classical activation markers or T-cell cytokines, the effect appears innate and phagocyte-intrinsic.	Protection (TB)	DOI: 10.1159/000512279
Plasmodium spp.	Chukwuanukwu et al. [177]	Human samples	2017	*Plasmodium spp.* → TB, TB → Plasmodium spp.	To evaluate how malaria coinfection influences cytokine responses to PPD in TB patients.	PBMCs from 231 participants were stimulated with PPD; cytokines (IFN-γ, IL-4, IL-10) and CD4 counts were analyzed in TB-only vs. TB + malaria/HIV groups.	*Plasmodium spp.* (not specified)	Single time-point sampling; stimulation of PBMCs for 72 h	Higher proinflammatory cytokines in LTBI were found to confer immunological protection against severe malaria, while weakening response to TB.	Exacerbation (TB)/Protection (*Plasmodium spp.*)	DOI: 10.1111/cei.12861
Plasmodium spp.	Hawkes et al. [154]	Cellular model	2010	*P. chabaudi* → TB	To investigate whether malaria infection exacerbates mycobacterial infection, and to characterize the underlying immunological mechanisms.	*In vitro*: murine macrophages infected with *Mtb*, followed by *P. chabaudi* exposure, before evaluation of bacterial load and macrophage responses.	*Plasmodium chabaudi chabaudi AS*	24 h after *Mtb* exposure, macrophages are incubated with parasitized erythrocytes for 1, 3, 5 and 7 days.	Malaria coinfection significantly increased mycobacterial loads *in vitro*.	Exacerbation (TB)	DOI: 10.1016/j.micinf.2010.05.013
Plasmodium spp.	Mueller et al. [196]	Mice model	2012	*P. berghei* → TB	To assess whether natural malaria infection exacerbates chronic *Mtb* in mice.	Mice chronically infected with *Mtb* were exposed to *P. berghei* via mosquito bite (natural transmission) or injection. Disease progression assessed via TB bacterial loads, cytokine profiling, histopathology and parasitemia.	*Plasmodium berghei* NK65 strain	Mice infected with *P. berghei* by mosquito bites 40 days after *Mtb* aerosol infection. Follow up to 13 days after *P. berghei* infection.	Mice co-infected with *Mtb* and *P. berghei* exacerbates chronic TB but are less susceptible to parasitic infection (lower parasitemia in peripheral blood and weight loss).	Exacerbation (TB)/Protection (*Plasmodium spp.*)	DOI: 10.3791/50829

Plasmodium spp.	Page et al. [195]	Mice model	2005	TB → *P. yoelii*	To test whether prior or ongoing *Mtb* infection impacts the severity of lethal malaria, possibly through potentiation of type 1 immune responses.	Mice infected with *Mtb* via aerosol prior to lethal *P. yoelii* injection. Compared parasitemia, survival, splenic cytokine production (IFN-γ, TNF-α), and host genetics (C57BL/6 versus BALB/c strains).	*Plasmodium yoelii* 17XL	*P. yoelii* infection 2 or 8 weeks post-*Mtb* infection. Measurements taken at days 1, 3, and 5 post-parasite challenge.	Coinfected mice displayed significantly lower peak parasitemia and improved survival compared to malaria-only controls. Splenocytes produced higher IFN-γ and TNF-α, with marked type 1 response potentiation at day 5. Protection could be mouse-strain specific as only C57BL/6 gained protection.	Protection (*Plasmodium spp.*)	DOI: 10.1128/IAI.73.12.8369-8380.2005
